# Unraveling oxygen vacancy site mechanism of Rh-doped RuO_2_ catalyst for long-lasting acidic water oxidation

**DOI:** 10.1038/s41467-023-37008-8

**Published:** 2023-03-14

**Authors:** Yi Wang, Rong Yang, Yajun Ding, Bo Zhang, Hao Li, Bing Bai, Mingrun Li, Yi Cui, Jianping Xiao, Zhong-Shuai Wu

**Affiliations:** 1 State Key Laboratory of Catalysis, Dalian Institute of Chemical Physics, Chinese Academy of Sciences, 457 Zhongshan Road, 116023 Dalian, China; 2 Dalian National Laboratory for Clean Energy, Chinese Academy of Sciences, 457 Zhongshan Road, 116023 Dalian, China; 3 University of Chinese Academy of Sciences, 19 A Yuquan Road, Shijingshan District, 100049 Beijing, China; 4 Department of Chemistry, School of Science, Tianjin University, 300072 Tianjin, China; 5 CAS Key Laboratory of Science and Technology on Applied Catalysis, Dalian Institute of Chemical Physics Chinese Academy of Sciences, 457 Zhongshan Road, 116023 Dalian, China; 6 Vacuum Interconnected Nanotech Workstation, Suzhou Institute of Nano-Tech and Nano-Bionics, Chinese Academy of Sciences, 215123 Suzhou, China; 7 School of Nano Technology and Nano Bionics University of Science and Technology of China, 230026 Hefei, China

**Keywords:** Electrocatalysis, Electrocatalysis, Hydrogen energy

## Abstract

Exploring durable electrocatalysts with high activity for oxygen evolution reaction (OER) in acidic media is of paramount importance for H_2_ production via polymer electrolyte membrane electrolyzers, yet it remains urgently challenging. Herein, we report a synergistic strategy of Rh doping and surface oxygen vacancies to precisely regulate unconventional OER reaction path via the Ru–O–Rh active sites of Rh-RuO_2_, simultaneously boosting intrinsic activity and stability. The stabilized low-valent catalyst exhibits a remarkable performance, with an overpotential of 161 mV at 10 mA cm^−2^ and activity retention of 99.2% exceeding 700 h at 50 mA cm^−2^. Quasi in situ/operando characterizations demonstrate the recurrence of reversible oxygen species under working potentials for enhanced activity and durability. It is theoretically revealed that Rh-RuO_2_ passes through a more optimal reaction path of lattice oxygen mediated mechanism-oxygen vacancy site mechanism induced by the synergistic interaction of defects and Ru–O–Rh active sites with the rate-determining step of *O formation, breaking the barrier limitation (*OOH) of the traditional adsorption evolution mechanism.

## Abstract (web summary)

Exploring highly active and durable Ru-based electrocatalysts for acidic water oxidation is challenging. Here authors reported an ion-exchange adsorption strategy to regulate oxygen vacancies and Rh dopant, with a corresponding fundamental investigation on the lattice oxygen oxidation and the oxygen vacancy site.

## Introduction

Oxygen evolution reaction (OER) plays an undoubtedly vital role in the energy conversion system, which involves hydrogen production through water electrolysis, CO_2_ reduction to generate clean small molecule fuel, and the application of energy conversion devices such as metal-air batteries^1–4^. As a key half-reaction, OER needs higher energy to overcome the kinetic barrier due to the four-electron transfer reaction^5–7^. Compared with an alkaline medium, OER under an acidic electrolyte presents a higher reaction rate, fewer side reactions, and a more complete electrolytic cell system design during electrochemical water splitting, owing to the fully developed polymer electrolyte membrane (PEM) reduces gas crossover and provides high proton conductivity, and more importantly, hydronium ions have higher conductivity (350 S cm^2^ mol^−1^) compared with hydroxide ions (198 S cm^2^ mol^−1^) for large current density^8,9^. Therefore, it is voracious to exploit cost-effective alternative OER electrocatalysts with high activity and durability in an acidic medium.

Currently, rutile-structured ruthenium (Ru) and iridium (Ir) oxides catalysts are widely used to improve the catalytic performance of acidic OER^10–12^. Notably, RuO_2_ has higher activity and lower price than IrO_2_, being considered as a most potential acidic OER catalyst^13,14^. However, RuO_2_ is limited by poor durability as a result of the collapse of active site structure and dissolution of Ru leading to deactivation under harsh acid conditions^15–18^. So far, it is generally accepted that the acidic OER mechanisms include the adsorption evolution mechanism (AEM) and the lattice oxygen mediated mechanism (LOM)^19,20^. The LOM can bypass the thermodynamic limitations of the typical AEM to enhance OER kinetics^21,22^. Recently, a series of LOM-based catalysts, including Si-SrCoO^21^, Zn_0.2_-Co_0.8_OOH^23^, and Ba_0.35_Sr_0.65_Co_0.8_Fe_0.2_O_3_-δ^24^, were explored to improve electrochemical performance toward OER in alkaline electrolytes by modulating the electronic structure of surface and interface. However, LOM is not as stable as AEM in acid because the lattice oxygen oxidation leads to severe surface remodeling of the catalyst, followed by dissolution in the electrolyte, affecting catalytic stability^8,25^. To achieve a balance between activity and stability, strategies of alloying^26,27^, defect engineering^28,29^, and heterogeneous element doping^30,31^ are developed to promote rate-determining step (RDS) *OOH formation of AEM or prevent excessive oxidation of Ru-based materials into soluble Ru^x+^ (x > 4) derivatives (e.g., RuO_4_) for LOM, simultaneously enhancing the activity and stability in acidic electrolyte. Among them, oxygen vacancies (O_V_) in oxides can effectively change the coordination environment of the metal active center, expose more active sites, and enhance the intrinsic catalytic activity, which can be created by hydrogen reduction, high-temperature calcination, or low-valent element doping methods^32,33^. As we demonstrated previously, intrinsically defective RuO_2_ via graphene oxide (GO) confined oxygenation strategy showed impressive OER activity but unfortunate stability^29^. Taken together, chemically stable O_V_ and low-valent active center are crucial to stabilizing the catalyst, thereby maintaining the long-term stability of the acidic OER process.

Inspired by the aforementioned design rationales, we herein develop Rh doping in RuO_2_ lattice on graphene (Rh-RuO_2_/G) with ion-exchange adsorption strategy taking GO as the confined oxygenation template, yielding stable O_V_ for compensating the effective negative charge of doped low-valent cations. More importantly, the rich O_V_ surface sites also play key roles in the activity, including the O 2p band center is close to the Fermi level, which can accelerate charge transfer, while catalyst surface adsorption optimization achieves a lower reaction energy barrier and higher H_2_O activation rate^34^. In addition to the greatly enhanced catalytic activity, the doping of Rh provides stable O_V_ for durable catalysis since it stabilizes the chemical coordination environment of the low-valent metal active sites for the OER process, proven by quasi in situ X-ray photoelectron spectroscopy (XPS) and ex-situ X-ray absorption fine structure (XAFS). Impressively, the enriched O_V_ in the as-fabricated Rh-RuO_2_/G nanosheets exerted a decisive effect on OER activity and stability in acidic media. Specifically, the Rh-RuO_2_/G nanosheets display remarkable OER performance with an overpotential of 161 mV and 214 mV at a current density of 10 mA cm^−2^ (*η*_10_) and 100 mA cm^−2^ (*η*_100_), TOF of 1.74 s^−1^ at an overpotential of 300 mV, and long-durable stability of 700 h, suppressing most active OER electrocatalysts reported to date. Density functional theory (DFT) demonstrates that Rh-RuO_2_/G passes through a more optimal reaction path of the lattice oxygen mediated mechanism-oxygen vacancy site mechanism (LOM-OVSM) for activity, further elaboration of the stabilization mechanism. Particularly, the Rh-RuO_2_/G catalyst with excellent intrinsic structure also exhibits promising performance for Li-O_2_ batteries, showing a small charge overpotential of 0.27 V and sustaining cyclability of 4500 h. Overall, the synergistic mechanism of Ru–O–Rh sites and stable O_V_ shed new insight for rationally fabricating high-performance metal oxide catalysts.

## Results

### Synthesis and characterization of Rh-RuO_2_/G

**Fig. 1:**
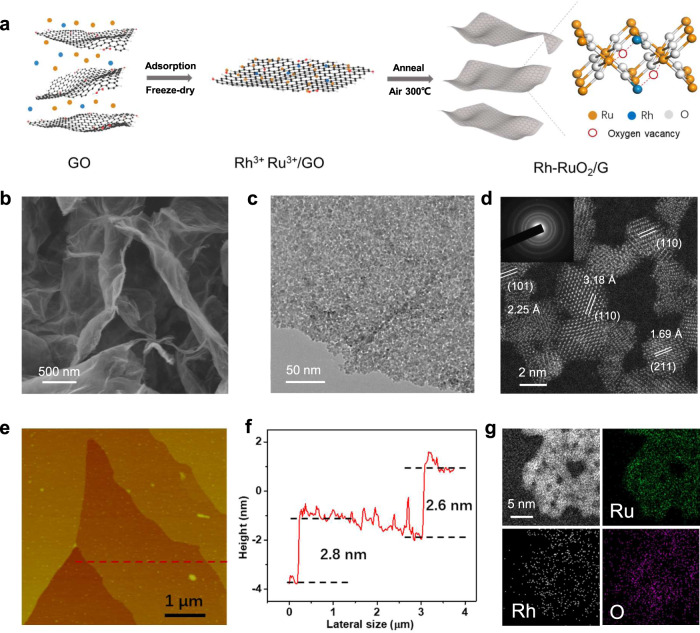
Synthesis and morphology characterization of Rh-RuO_2_/G nanosheets. **a** Preparation schematic. **b** SEM image. **c** TEM image. **d** HAADF-STEM image (inset shows the SEAD pattern). **e**, **f** AFM image (**e**) and corresponding height profiles (**f**). **g** Elemental mapping analysis of Rh-RuO_2_/G.

The Rh-RuO_2_/G catalyst was synthesized following the ion-adsorption approach^29^, as illustrated in Fig. 1a. The Rh doping amount is 1.38 wt% in the RuO_2_ matrix (Supplementary Table 1). Scanning electron microscopy (SEM, Fig. 1b) and transmission electron microscopy (TEM, Fig. 1c) images demonstrate the flexible laminar morphology of the as-prepared Rh-RuO_2_/G with smooth surfaces and evenly dispersed nanostructures. The average thickness of Rh-RuO_2_/G nanosheets is measured to be 2.7 nm, as identified by atomic force microscopy (AFM, Fig. 1e, f), thereby exposing more inner layer atoms and providing effective active sites for catalytic reactions. The high-angle annular dark-field scanning TEM (HAADF-STEM) image in Fig. 1d shows the interconnecting small-size nanocrystals (≈2 nm) and lattice fringes corresponding to the (110), (101), and (211) planes for rutile-structured RuO_2_. Moreover, the selective electron diffraction (SAED) image indicates the polycrystalline nature of Rh-RuO_2_/G (inset in Fig. 1d). Elemental mapping analysis (Fig. 1g) confirms homogeneous distribution of Ru, Rh, O, and C signals throughout the entire nanosheet.

**Fig. 2:**
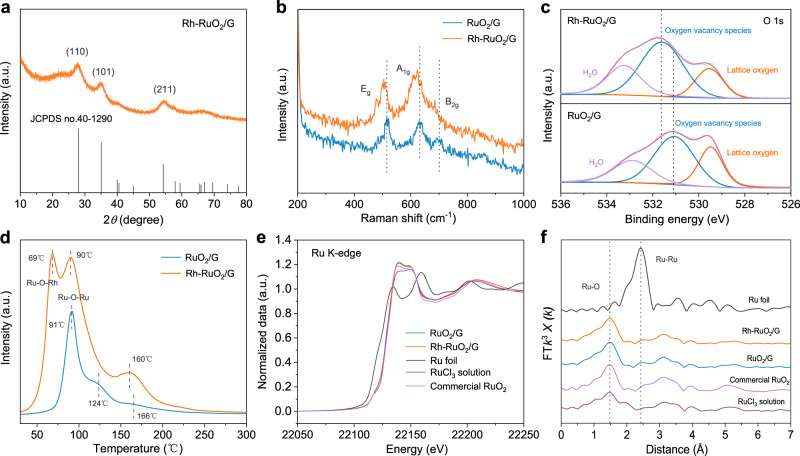
Compositional structure analysis of Rh-RuO_2_/G nanosheets. **a** XRD pattern of Rh-RuO_2_/G. **b** Raman spectra, **c** *O 1s* XPS spectra, and **d** H_2_-TPR spectra of Rh-RuO_2_/G and RuO_2_/G. **e** Ru K-edge XANES spectra and **f** Fourier transformed EXAFS spectra of Ru-edge for Rh-RuO_2_/G, RuO_2_/G, Ru foil, RuCl_3_ solution, and commercial RuO_2_.

The crystal structure of the as-obtained Rh-RuO_2_/G was validated by X-ray diffraction (XRD) pattern (Fig. 2a), in which three prominent broadened diffraction peaks are well matched with the (110), (101), and (211) facets of rutile-structured RuO_2_ (JCPDS no.40-1290), indicative of complete conversion of Ru^3+^ into small RuO_2_. Electron paramagnetic resonance (EPR), as strong evidence for characterizing unpaired electrons, is used to explore the generation and variation of O_V_. Significantly, Rh-RuO_2_/G shows a significant EPR signal at *g*_e_ = 2.003 (Supplementary Fig. 1) due to O_V_ capturing unpaired electrons^35^. Raman spectrum shows the nanocrystalline nature of RuO_2_ (Fig. 2b), with three distinctive peaks centered at 516, 633, and 700 cm^−1^ corresponding to the *E*_g_, *A*_1g_, and *B*_2g_, respectively. After Rh doping, the peaks appear broadening and blue shift, signifying that local incorporation transforms the phonon vibration frequency by weakening symmetry and higher oxygen vacancy concentration promotes Ru–O vibration frequency^36^. It is noteworthy that the *B*_2g_ peak disappears in Rh-RuO_2_/G, which is attributed to the location of Rh(III) at the position of Ru(IV) sites in octahedral units by cations exchange rather than surface adsorption^37,38^. Figure 2c compares the *O 1s* XPS to verify the defective structure. It can be seen that three well-fitted peaks are well deconvoluted into the lattice oxygen (Ru–O) at 529.5 eV, O_V_ at 531.2 eV, and adsorbed water (H_2_O) at 533 eV, while the O_V_ peaks for Rh-RuO_2_/G clearly shift to higher binding energy, proving the existence and chemical environment change of O_V_^39^. Significantly, the variation in the proportion of surface oxygen vacancy species and lattice oxygen (O_V_/O_L_) is observed from 1.76 to 2.86 after doping, signifying an abundance of O_V_ and thus excellent electronic capture and transfer properties of the nanosheets. It is understandable that O_V_ are generated to maintain charge neutrality after exchanging a low oxidation state of Rh. The *Ru 3p* XPS spectrum (Supplementary Fig. 2a) exhibits two predominant peaks located at 484.8 and 462.7 eV, slightly lower than the binding energy of Ru (IV)^40^. Similarly, the lower binding energy in the *Rh 3d* XPS spectrum (Supplementary Fig. 2b) indicates the presence of low-valent Rh species, corresponding to 308.6 and 313.6 eV of anoxic rhodium oxides^41^. The H_2_ temperature-programmed reduction (H_2_-TPR, Fig. 2d) profile of RuO_2_/G exhibits a predominant peak located at 90 °C, corresponding to the conversion of Ru^4+^ to metallic Ru, which is lower than the reduction temperature of commercial RuO_2_ (213 °C) due to the small particle size^42^. The relatively weak peaks positioned at 100–200 °C can be ascribed to the variation in the particle sizes. In addition, Rh-RuO_2_/G shows a lower reduction peak at 69 °C as a result of the relatively weak asymmetric oxygen species (Ru–O–Rh), revealing the existence of an active center that requires further verification by DFT^43,44^. To further reveal the coordination structure and bond configurations of as-prepared Rh-RuO_2_/G, X-ray absorption spectroscopy (XAS) was employed to evaluate the valence state and coordination environment of Ru centers. The X-ray absorption near edge structure (XANES) of Ru K-edge region and the extended X-ray absorption fine structure measurements of Ru K-edge Fourier transformation (FT-EXAFS) are conducted with Ru foil, RuCl_3_ solution, and commercial RuO_2_ as references (Fig. 2e, f). Figure 2e shows that the absorption threshold of Rh-RuO_2_/G lies between Ru foil and commercial RuO_2_, closing to RuCl_3_ solution, visualizing the average valence state of Ru is between Ru(0) and Ru(IV)^45^, which is consistent with the XPS results. FT-EXAFS spectrum in Fig. 2f displays a main peak positioned at ~1.5 Å attributing to the backscattering of Ru–O in the first shell, while the Ru–Ru bond is at 2.4 Å^28,45^. The peak at 3.12 Å in Rh-RuO_2_/G originates from the backscattering of Ru–Ru and Ru–Rh in the second shell (Supplementary Fig. 4), exhibiting lower vibrational amplitudes than standard RuO_2_, which is ascribed to the small size of nanoparticles^13,46^. Besides, the Ru–O peak intensity of Rh-RuO_2_/G is slightly lower than standard RuO_2_, owing to the insufficient coordination of Ru. Compared with pure RuO_2_, the lower coordination number of 5.7 for Ru–O in Rh-RuO_2_/G is identified by EXAFS fitting results (Supplementary Table 2) as a result of O_V_, laying the foundation for theoretical computational modeling and mechanism analysis.

### Electrocatalysis of OER

**Fig. 3:**
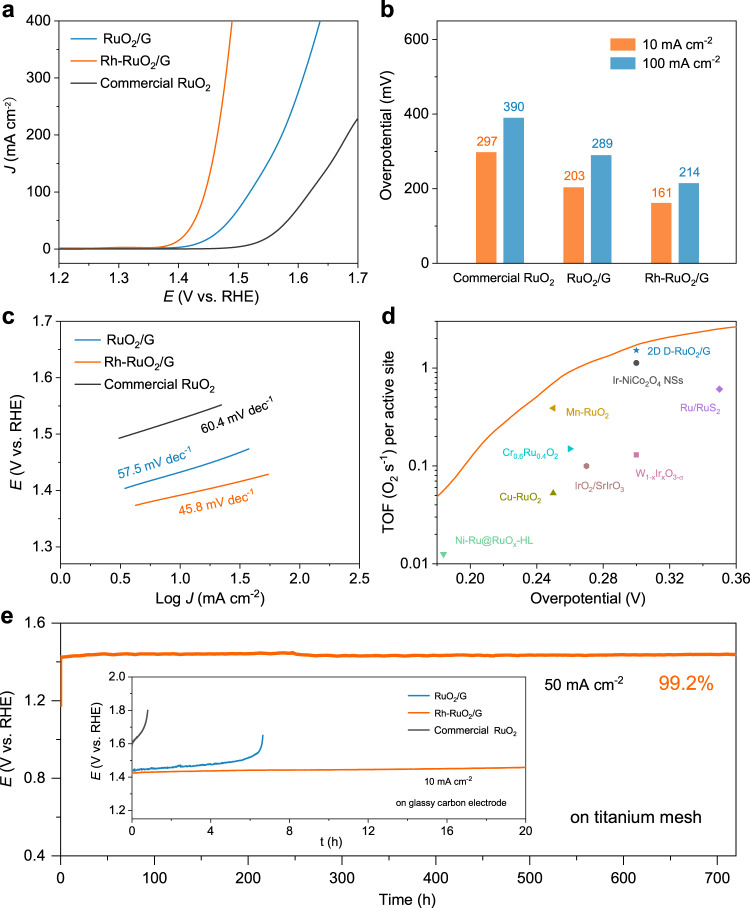
Electrocatalytic performance of Rh-RuO_2_/G, RuO_2_/G, and commercial RuO_2_ catalysts in 0.5 M H_2_SO_4_ solution with 95% *iR* compensation. **a** LSV polarization curves. **b** Overpotentials at a current density of 10 mA cm^−2^ and 100 mA cm^−2^. **c** Tafel plots. **d** The TOF curve of Rh-RuO_2_/G compared with reported representative catalysts. **e** The galvanostatic curves at a current density of 10 mA cm^−2^ and 50 mA cm^−2^.

To demonstrate the catalytic ascendancy of rich O_V_ with Rh doping, the OER performance of the Rh-RuO_2_/G catalyst was recorded in O_2_ saturated 0.5 M H_2_SO_4_ electrolyte. As shown by the linear sweep voltammetry (LSV) curves in Fig. 3a, the Rh-RuO_2_/G catalyst exhibits an *η*_10_ of only 161 mV, which is lower than as-fabricated RuO_2_/G (203 mV) and commercial RuO_2_ (297 mV), suggesting that the defects and Ru–O–Rh active center are conducive to acidic OER process with excellent performance. Moreover, our Rh-RuO_2_/G presents a significant small overpotential of *η*_100_ = 214 mV (Fig. 3b). Further, the Tafel slope of Rh-RuO_2_/G nanosheets is ≈45.8 mV dec^−1^, smaller than those of RuO_2_/G (57.5 mV dec^−1^), and even the benchmark RuO_2_ (60.4 mV dec^−1^) (Fig. 3c). Such a low Tafel slope value signifies the fast kinetic merit of Rh-RuO_2_/G nanosheets for water oxidation. As depicted in Fig. 3d, the turnover frequencies (TOF) value corresponding to the intrinsic per site activity of the as-obtained Rh-RuO_2_/G is 1.74 O_2_ s^−1^ at an overpotential of 300 mV, which is considerably higher than most reported catalysts^8,9,13,14,30,47–51^. Additionally, the electrochemical double-layer capacitance (*C*_dl_) values, extracted from cyclic voltammetry (CV, Supplementary Fig. 7), are closely associated with electrochemically active surface areas (ECSAs) of catalyst. Rh-RuO_2_/G displays noticeably larger *C*_dl_ and ECSA values (110.6 mF cm^−2^, 2765 cm^2^) than RuO_2_/G (79.3 mF cm^−2^, 1982.5 cm^2^) and commercial RuO_2_ (10.9 mF cm^−2^, 272.5 cm^2^) (Supplementary Fig. 6). It is indicated that Rh-RuO_2_/G nanosheets expose abundant active sites for facilitating OER activity. The electrochemical stability was evaluated by galvanostatic electrocatalysis. Obviously, Rh-RuO_2_/G maintains almost unchanged performance (99.2%) to the initial state for 700 h at a constant current density of 50 mA cm^−2^, superior to RuO_2_/G and commercial RuO_2_ (Fig. 3e). The stability is further demonstrated by a long-term operation of 500 h at a higher current density of 100 mA cm^−2^ (Supplementary Fig. 8). Interpretably, the poor stability of RuO_2_/G originates from unrestricted O_V_ and being vulnerable to destruction during the sustained OER process, while the doping of Rh creates a steady bulk O_V_ structure and low-valent metal center for long-term durability. Therefore, our Rh-RuO_2_/G catalyst is one of the best acidic OER catalysts in terms of excellent overpotential and durability (Supplementary Table 3). To highlight the applicability for energy conversion, a two-electrode electrolyzer with Rh-RuO_2_/G as anode and Pt/C as cathode was performed for overall water splitting in acidic media. Remarkably, the polarization curve for water splitting exhibits a low cell voltage of 1.42 V at 10 mA cm^−2^ and maintains a pretty stable performance (Supplementary Fig. 9).

### Structural analysis for enhanced activity and durability

**Fig. 4:**
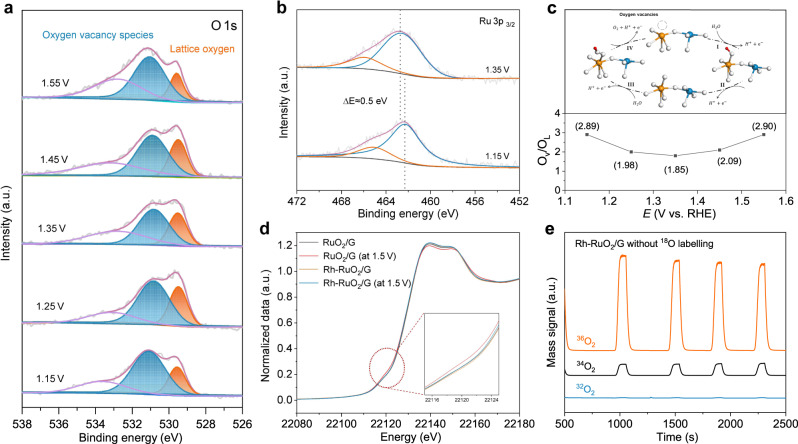
Quasi in situ NAP-XPS, ex-situ XAFS, and operando DEMS measurements probing dynamic evolution of electronic structure on Rh-RuO_2_/G catalyst during OER reaction. **a**, **b** The quasi in situ *O 1s* (**a**) and *Ru 3p*_*3/2*_ (**b**) XPS spectra recorded of the resultant Rh-RuO_2_/G during the multipotential steps. **c** Variation of O_V_/O_L_ ratio from quasi in situ XPS measurement. **d** Ex-situ XANES spectra for Rh-RuO_2_/G and RuO_2_/G at 1.5 V for 8 h during OER. Inset: magnified pre-edge XANES region. **e** DEMS signals of ^36^O_2_, ^34^O_2_, and ^32^O_2_ for Rh-RuO_2_/G in H_2_^18^O aqueous sulfuric acid electrolyte within four times of LSV at 1.1–1.9 V (vs. RHE).

To probe the evolution of electronic structure at the surface of the catalysts, we carried out quasi in situ XPS studies to examine the potential-dependent oxidation of *O 1s* and *Ru 3p*_*3/2*_ during the chronoamperometry test for OER, monitored by the analysis chamber of the in situ near ambient pressure XPS (NAP-XPS) system. Figure 4a shows the variation in the proportion of surface oxygen vacancy species and lattice oxygen with the potential scanned from 1.15 to 1.55 V. A high ratio of O_V_/O_L_ implies preferential adsorption of water at the intrinsic O_V_ sites due to steric hindrance, which is closely connected with optimizing adsorption and desorption of intermediate oxygen species. Moreover, at higher potentials (1.55 V), the O_V_/O_L_ ratio almost returns to the initial state (2.89), manifesting that O_V_ regeneration is accompanied by the release of oxygen to endow the stabilization of the active site structure (Fig. 4c)^52^. Similarly, the *Ru 3p*_*3/2*_ peak position in Rh-RuO_2_/G shifts to higher binding energy (*ΔE* = 0.5 eV) at 1.35 V (Fig. 4b) since lower electron density around Ru atom after the lattice oxygen dominates. The phenomenon of cycling of reversible oxygen species demonstrates the priority of the LOM-OVSM, LOM-SMSM (the lattice oxygen mediated mechanism-single metal site mechanism). In order to further verify that lattice oxygen is not involved in OER, operando ^18^O isotope labeling differential electrochemical mass spectrometry (DEMS) measurements were conducted. Figure 4e shows the main mass signals of ^36^O_2_, followed by ^34^O_2_ and ^32^O_2_, for the Rh-RuO_2_/G without ^18^O-labeling during four times of LSV in H_2_^18^O aqueous sulfuric acid electrolyte, where trace amounts of ^34^O_2_ product was attributed to the natural isotopic abundance of ^18^O water～2 at.%. It is implied that the lattice oxygen atom is not participated in oxygen production, thereby excluding the traditional LOM. Consistently, a large number of ^32^O_2_ product was detected without any ^36^O_2_ for the ^18^O-labeled Rh-RuO_2_/G catalyst in H_2_^16^O aqueous sulfuric acid electrolyte (Supplementary Fig. 10). The quasi in situ XPS and DEMS results lay a foundation for theoretically simulating optimal reaction paths.

Meanwhile, the stability of Rh-RuO_2_/G under working conditions was verified by operando Raman characterization techniques. As shown in Supplementary Fig. 11, it is evidenced that the crystal structure keeps almost unchanged, as seen from the Raman vibrational displacement at the voltage of 1.05–1.9 V. To further explore the source of the stability difference, the local geometry evolution of Ru was observed by XANES under ex-situ conditions (Fig. 4d). Compared with the stable absorption line of Rh-RuO_2_/G at initial and 1.5 V, RuO_2_/G depicts an obvious characteristic pre-edge 1s-4d transition after reaction (around 22120 eV), and the intensity of this shoulder peak is positively correlated with the unoccupied Ru 4d state^53,54^. It is disclosed that more electrons are transferred from 4d to nearby atoms, resulting in the increase of the average valence state of Ru, associated with additional oxygen species adsorption^6,55^. Therefore, it is inferred that the deactivation of RuO_2_/G is caused by the distortion of the coordination geometry and the weak change of the Ru oxidation state^27,56^. Overall, benefiting the stabilizing enriched O_V_ and low-valent active sites (Ru–O–Rh), the Rh-RuO_2_/G catalyst performs excellent activity and durability.

### Theoretical insights on intrinsic activity and stability

DFT calculations were performed to understand the chemical origin of excellent OER activity and stability over Rh-RuO_2_/G. RuO_2_ (110), (211), and (101) perfect (‘p’) surfaces, three dominant peaks in the XRD pattern (Fig. 2a), were chosen as possible active surfaces. The defect RuO_2_ surfaces (‘O_V_’) and Rh doping RuO_2_ surfaces (‘d’) were also calculated for comparison. Correspondingly, the ‘d-O_V_’ denotes the defect RuO_2_ surfaces with Rh doping. Based on previous studies^57,58^, three OER mechanisms were considered, as shown in Supplementary Fig. 14, namely, AEM and two evolutionary lattice oxygen mediated mechanisms, LOM-OVSM and LOM-SMSM. For LOM-OVSM mechanism, a water molecule first deprotons to produce *OH at the O_V_ site next to the doped Rh site, resulting in Ru–OH bond, followed by deprotonation and *O formation. Then, another water deprotons and produce an *OOH. Finally, as the final deprotonation occurs, an O_2_ molecule can be evolved and desorbed from the surface, simultaneously reproducing the O_V_ again.

**Fig. 5:**
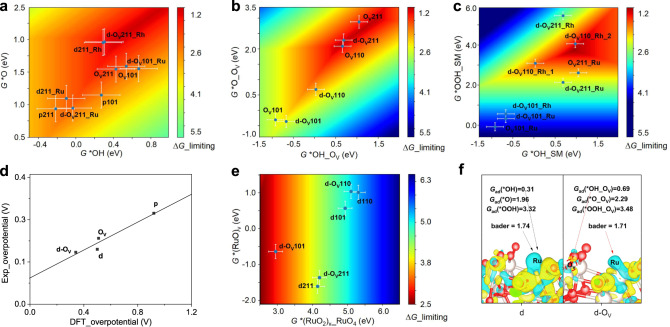
DFT calculation of the OER reaction mechanism to elucidate intrinsic activity and stability of Rh-RuO_2_. **a**–**c** A two-dimensional activity map for AEM mechanism (**a**), LOM-OVSM mechanism (**b**), and LOM-SMSM (**c**) mechanism based on OER. The adsorption energies of *OH (*OH_O_V_ or *OH_SM) and *O (*O_O_V_ or *O_SM) are applied to be two independent descriptors. A 0.2 eV error bar is shown to describe the theoretical uncertainty. **d** Comparisons between calculated overpotential and experiment overpotential relationship. **e** A two-dimensional stability map for d and d-O_V_. **f** Charge density difference analysis for d and d-O_V_ slab.

According to the scaling relations between the adsorption free energies (Supplementary Figs. 15–17), the adsorption free energies of *OH and *OH_O_V_ and *OH_SM are used as the primary descriptors of the reaction free energies (*ΔG*) of all elementary steps. Furthermore, the adsorption free energies of *O, *O_O_V_, and *OOH_SM are introduced as another dimension to build the two-dimensional activity maps of different mechanisms, as shown in Fig. 5a–c. DFT calculated free energy diagrams are shown in Supplementary Fig. 18, where the activities (overpotentials) over different surfaces are compared. It is found that the *OOH formation limited its overall activity in the perfect RuO_2_ and defect RuO_2_ surfaces, while the potential-determining step of defect Rh-RuO_2_ is the formation of *O (0.34 eV) by the synergistic effect of Rh doping and O_V_. Therefore, the activity of the examined catalysts is predicted to be defect Rh-RuO_2_ > defect RuO_2_ > perfect RuO_2_, with the distinctive mechanisms, namely, LOM-OVSM, LOM-OVSM, and AEM, respectively. A quantitative comparison between theoretical overpotentials and experimental ones exhibits a good correlation, which confirms our calculations and analysis above regarding the active site and reaction mechanism (Fig. 5d). It is indicated that the OER over the d-O_V_211 facet of the experimental Rh-RuO_2_/G samples is likely to follow the LOM-OVSM, which is attributed to the synergistic interaction of O_V_ with the nearby metal site, consistent with the results observed by quasi in situ XPS.

As reported previously^59^, it is proposed that the stability loss of OER catalysts is induced by the dissolution of RuO_4_ species; the catalyst is gradually deactivated in this process (see the proposed mechanism in Supplementary Fig. 19). To understand the stability of Rh-RuO_2_, the dissolution energy of RuO_4_ species was calculated. The stability of various active sites is shown in Fig. 5e, where the blue area denotes better stability. Although the d-O_V_211 facet of Rh-RuO_2_ exhibits moderate stability, compared to d-O_V_110 and d-O_V_101, d-O_V_211 should exhibit a dominant contribution to OER performance, considering both activity and stability. Furthermore, to interpret the activity and stability of d-O_V_211, we compared the electronic structures of Rh-doped case and d-O_V_211 based on the charge density difference analysis. As O_V_ are introduced, the modified site (d-O_V_211) becomes a better active center with lower electron density, resulting from the appropriate binding energy of reacting species, which is the fundamental reason for the improved activity of Rh-RuO_2_. In addition, as depicted in Fig. 5f, the electronic density on the surface Ru between d211 and d-O_V_211 is comparable (1.74 vs. 1.71) based on Bader charge analysis, showing that the stability was not reduced. Hence, the observed OER stability can be understood well by DFT results.

As for the contribution of RhO_2_ for OER, the large-size RhO_2_ nanocrystals can be excluded by the XRD pattern of Rh/G (Supplementary Fig. 5). DFT calculations were performed to investigate the contribution of OER activity from RhO_x_ clusters as the adsorption energies are site- and size-dependent. Rh_6_O_6_ and Rh_6_O_12_ clusters were considered, supported on the most stable RuO_2_(101) facet, and two sites for all the clusters. Further, we considered both perfect and defective RuO_2_(101) as substrates. At some sites, the activity of RhO_x_ clusters is indeed great (Supplementary Table 8). For instance, the sites of Rh_6_O_6_ cluster on defect RuO_2_(101) substrate show similar overpotential (0.32 V vs. RHE) compared with the defect RuO_2_ surfaces with Rh doping (0.34 V vs. RHE). Therefore, the contribution from the intrinsic activity of small RhO_x_ clusters cannot be strictly ruled out^57^. It is noted that RhO_x_ clusters should be dominated by high oxidation states, as the limited oxygen vacancies on pure RuO_2_ under OER condition. In other words, the Rh_6_O_12_ supported by perfect RuO_2_(101) should be the major case compared to the others, whose overpotentials are pretty large (1.67 V and 1.93 V vs. RHE). Besides, all the studied facets of Rh-RuO_2_ are more stable compared to individual RhO_2_ (Supplementary Table 10). By the combined analysis of experimental and computational results (both activity and stability), the Rh element should be primarily present as Rh-RuO_2_.

In addition to the acidic OER performance, Rh-RuO_2_/G also performs impressive full water splitting catalytic performance with a cell voltage of 1.45 V and long-term stability in alkaline electrolyte, significantly exceeding RuO_2_/G and commercial RuO_2_ (Supplementary Fig. 12). It is suggested that the intrinsic defect structure and rich active sites working together optimize the adsorption activation process of the intermediate to reduce the overpotential of the electrocatalytic reaction. Not only that, we leverage the OER electrocatalytic activity of Rh-RuO_2_/G as cathode catalysts in Li-O_2_ battery (Supplementary Fig. 13), showing efficiently reduced charge overpotential of 0.27 V and long-term stability with a long cycle life of 4500 h, further illustrating the potential of this material in diversified energy catalysis and conversion systems.

## Discussion

In summary, we demonstrate a synergetic regulation strategy of Rh doping and surface oxygen vacancies in stabilizing low-valent Ru of RuO_2_ catalyst, arousing a LOM-OVSM pathway for impressive active and durable OER in harsh acidic media. Our stabilized Rh-RuO_2_/G catalyst shows a low overpotential of *η*_10_ = 161 mV and long-term stability for 700 h at 50 mA cm^−2^, surpassing most reported noble metal-based catalysts. It is validated that the presence of reversible oxygen vacancies improves the intrinsic activity and crystal structure stability under working potentials verified by quasi in situ*/*operando characterization techniques. DFT reveals that the Ru–O–Rh site with defect induces LOM-OVSM mechanism for extraordinary activity and durability. The formation of *O is the RDS of Rh-RuO_2_/G, breaking the AEM-limited reaction barrier (*OOH) of the conventional RuO_2_ system. This work suggests new inspiration to design high-performance acidic OER catalysts and in-depth mechanistic analysis for practical application in PEM electrolyzers.

## Methods

### Chemicals

Ruthenium (III) chloride trihydrate (RuCl_3_·3H_2_O), Rhodium (III) chloride (RhCl_3_), and commercial RuO_2_ were purchased from Aladdin Co., Ltd. The GO solution (6.2 mg ml^−1^) was synthesized via the modified Hummers’ method.

### Preparation of Rh-RuO_2_/G

First, 5 ml GO solution (6.2 mg ml^−1^) was diluted with 26 ml water, then mixed with 15 mg RuCl_3_·xH_2_O and 1 mg RhCl_3_ to form a homogeneous solution. Further, the mixture was frozen with liquid nitrogen and vacuum freeze drying for 72 h. Finally, the as-obtained powder was annealed with air under 300 °C for 3 h, leading to the formation of Rh-RuO_2_/G. For comparison, RuO_2_/G nanosheets were synthesized according to a similar protocol without adding RhCl_3_; Rh/G nanosheets were synthesized according to a similar protocol without adding RuCl_3_.

### Materials characterization

XRD was performed by a SmartLab with Cu Kα radiation. SEM images were acquired on a JSM7900F. TEM, HRTEM, HAADF-STEM images, and elemental mapping images were detected by an HT7700 microscope and ARM300 microscope with spherical aberration corrector. AFM images were conducted on a MultiMode 3D. XPS spectra were recorded on Hermo Scientific K-Alpha. ICP-AES was measured in PerkinElmer Optima 7300DV for quantifying metal content in the catalysts. EPR was carried out on a Bruker A200 at 77 K. Raman spectroscopy was recorded by LabRAM HR 800 with an excitation laser of 532 nm. The N_2_ sorption isotherms and Brunauer–Emmett–Teller (BET) surface areas were conducted by ASAP 2020 instrument at 77 K. H_2_-TPR experiments were performed with an AutoChem 2920 instrument. The XANES and EXAFS data were collected in the BL14W1 beamline of the Shanghai Synchrotron Radiation Facility (SSRF) under ambient air. EXAFS analysis, including calibration of the energy scale, correction of signal background, normalized signal intensity, and Fourier transform on *k*^2^-weighted EXAFS oscillations, was carried out on Athena software.

### Electrochemical measurements

Electrochemical measurements were evaluated in 0.5 M H_2_SO_4_ electrolyte (pH = 0.3) with a three-electrode configuration by a CHI 760E workstation. The cell contained a catalyst-modified glassy carbon electrode (GCE, 3 mm in diameter), Ag/AgCl filled with saturated KCl, and a graphite rod as the working electrode, the reference electrode, and the counter electrode, respectively. Furthermore, the well-dispersed catalyst loading on the GCE was ~0.45 mg cm^−2^. LSV curves were carried out at 5 mV s^−1^ with 95% *iR* drop compensation. As for the durability test of chronopotentiometry, 40 μl catalyst ink (5 mg ml^−1^) was evenly scribbled on the surface of the titanium mesh, and the mass loading density of all samples in titanium mesh was ~1.25 mg cm^−2^. Moreover, the same electrode fabrication method is used for water splitting in a two-electrode system. Potentials measured were converted to reversible hydrogen electrode (RHE) by the following equation: *E*(RHE) = *E*(Ag/AgCl) + 0.059 pH + 0.197.

### Quasi/in situ and operando characterizations

XPS measurements were performed using the SPECS NAP-XPS instrument attached to the glove box through a vacuum channel. The Rh-RuO_2_/G catalysts subjected to OER reaction at a range of potentials (1.05–1.55 V vs. RHE) were transferred to the analysis chamber utilizing a vacuum channel for further XPS measurements. Operando DEMS with isotope labeling measurements were carried out in an electrochemical cell with a typical three-electrode system using the QAS 100 device (Linglu Instruments, Shanghai). The Rh-RuO_2_/G catalyst was labeled with ^18^O isotopes by performing four times LSV cycles at 1.1–1.9 V (vs. RHE) with a scan rate of 5 mV s^−1^ in H_2_^18^O aqueous sulfuric acid electrolyte. Meanwhile, the mass signals of different molecular weight gas products were recorded in real time. After cleaning the surface of electrodes with H_2_^16^O, the same electrochemical and mass spectrometry were operated for ^18^O-labeled Rh-RuO_2_/G catalyst in H_2_^16^O aqueous sulfuric acid electrolyte. In situ Raman measurements were carried out in an in situ Raman cell equipped with a three-electrode system. Simultaneous acquisition of multipotential Raman spectra while catalyst OER procedure. Raman spectra were recorded by a LabRAM HR 800 Raman spectrometer with an excitation laser of 532 nm.

### Theoretical calculation methods

All calculations were performed by Vienna Ab initio Simulation Package (VASP)^60^ based on the DFT method, the generalized gradient approximation (GGA)^61^ of the Perdew–Burke–Ernzerhof (PBE)^62^ functional, and the projector-augmented wave (PAW)^63,64^ with a cutoff of 450 eV were applied. The perfect RuO_2_(p), Rh-doped RuO_2_(d), and the defective systems (O_V_ and d-O_V_) were all studied. The (110), (101), and (211) surfaces were chosen, respectively, for modeling the facets that were observed experimentally for nanocrystalline rutile oxides^28,65^. In addition, the surface energy of the mixed phase with respect to pure metal phases and individual oxide phases were studied, respectively (Supplementary Table 5 and Table 10). Furthermore, the effect of different amounts of Rh incorporation on the stability was tested (Supplementary Table 6). The Monkhorst-Pack scheme with the *k* points of (3×2×1) and (2×3×1) were applied for the (110)/(211) and (100) surfaces, respectively. All fix the bottom half of the atoms during optimization. In addition, a 20 Å vacuum layer along the *c*-axis was used to eliminate the interactions of periodic images. Dipole corrections were applied to account for possible dipole interactions between unit cells. The convergence criteria for energy and force were set to 10^−4^ eV and 0.03 eV Å^−1^, respectively. In addition, spin-polarization was considered. All energies calculated by DFT are corrected to the temperature of 298 K. Computational hydrogen electrode approximation was used to implicitly describe the chemical potential of a (H^+^ + e^−^) pair.

### Theoretical activity and stability analysis

The adsorption free energy of each intermediate was first calculated with reference to H_2_O(l) and H_2_(g). For example, the *OH is calculated as *G*(*OH) = *E*_ad_(*OH) + ZPE(*OH) − TS(*OH), where the *G*(*OH) was the adsorption free energy of intermediate *OH, the *E*_ad_(*OH) is the adsorption energy of *OH, the ZPE(*OH) is the zero-point energy of *OH, and the TS(*OH) is entropy correction, respectively (Supplementary Table 7). The scaling relationships of all adsorption free energies are shown in Supplementary Figs. 15–17. Then, the reaction energy (Δ*G*) of each elementary step, such as *OH → *O + (H^+^ + e^−^), can be calculated as Δ*G* = *G*(*O) + *G*(H^+^ + e^−^) − *G*(*OH), where *G*(*O) is the adsorption energy of *O and *G*(H^+^ + e^−^) is the chemical potential of the proton and electron pair at 0 V vs. RHE calculated according to the computational hydrogen electrode^66^. *G*(H^+^ + e^−^) was a constant under a certain voltage, and *G*(*O) was linearly correlated with *G*(*OH). Therefore, Δ*G* was also linearly scaled with *G*(*OH). As a result, the Δ*G* as a linear function of *G*(*OH) can be obtained. More details can be found in our previous work^67–70^.

### Theoretical stability analysis

As shown in Supplementary Fig. 19, the theoretical stability of the electrode is described by the reaction free energy (Δ*G*_limiting_) of the most thermodynamically difficult elementary step during the dissolution of ‘RuO_4_’ species^59^.

## Supplementary information

Supplementary Information

## Data Availability

The data generated in this study are provided in the main text and Supplementary information, where the source data of Figs. 2, 3, 4, and 5 are listed in the Source Data file. Extra data are available from the corresponding author upon reasonable request. Source data are provided with this paper.

## Source data

Source Data

## References

[1] Xia C, Jiang Q, Zhao C, Hedhili MN, Alshareef HN (2016). Selenide-based electrocatalysts and scaffolds for water oxidation applications. Adv. Mater..

[2] An L (2021). Recent development of oxygen evolution electrocatalysts in acidic environment. Adv. Mater..

[3] Sun Z (2021). Synergized multimetal oxides with amorphous/crystalline heterostructure as efficient electrocatalysts for lithium–oxygen batteries. Adv. Energy Mater..

[4] Zeng Z (2021). Orbital coupling of hetero-diatomic nickel-iron site for bifunctional electrocatalysis of CO_2_ reduction and oxygen evolution. Nat. Commun..

[5] Qin Q (2020). Gettering La effect from La_3_IrO_7_ as a highly efficient electrocatalyst for oxygen evolution reaction in acid media. Adv. Energy Mater..

[6] Cao L (2019). Dynamic oxygen adsorption on single-atomic ruthenium catalyst with high performance for acidic oxygen evolution reaction. Nat. Commun..

[7] Xu J (2021). Atomic-step enriched ruthenium–iridium nanocrystals anchored homogeneously on MOF-derived support for efficient and stable oxygen evolution in acidic and neutral media. ACS Catal..

[8] Zhu J (2021). Regulative electronic states around ruthenium/ruthenium disulphide heterointerfaces for efficient water splitting in acidic media. Angew. Chem. Int. Ed..

[9] Wen Y (2021). Stabilizing highly active Ru sites by suppressing lattice oxygen participation in acidic water oxidation. J. Am. Chem. Soc..

[10] Cui X (2020). Robust interface Ru centers for high-performance acidic oxygen evolution. Adv. Mater..

[11] Chen D (2020). Ionothermal route to phase-pure RuB_2_ catalysts for efficient oxygen evolution and water splitting in acidic media. ACS Energy Lett..

[12] Shi Z (2021). Confined Ir single sites with triggered lattice oxygen redox: toward boosted and sustained water oxidation catalysis. Joule.

[13] Lin Y (2019). Chromium-ruthenium oxide solid solution electrocatalyst for highly efficient oxygen evolution reaction in acidic media. Nat. Commun..

[14] Su, J. et al. Assembling ultrasmall copper-doped ruthenium oxide nanocrystals into hollow porous polyhedra: highly robust electrocatalysts for oxygen evolution in acidic media. *Adv. Mater.* **30**, 1801351 (2018). 10.1002/adma.201801351 29870585

[15] Chen F-Y, Wu Z-Y, Adler Z, Wang H (2021). Stability challenges of electrocatalytic oxygen evolution reaction: from mechanistic understanding to reactor design. Joule.

[16] Zhang Y, Zhu X, Zhang G, Shi P, Wang A-L (2021). Rational catalyst design for oxygen evolution under acidic conditions: strategies toward enhanced electrocatalytic performance. J. Mater. Chem. A.

[17] Moysiadou A, Lee S, Hsu CS, Chen HM, Hu X (2020). Mechanism of oxygen evolution catalyzed by cobalt oxyhydroxide: cobalt superoxide species as a key intermediate and dioxygen release as a rate-determining step. J. Am. Chem. Soc..

[18] Grimaud A (2017). Activating lattice oxygen redox reactions in metal oxides to catalyse oxygen evolution. Nat. Chem..

[19] Chen Z (2022). Advances in oxygen evolution electrocatalysts for proton exchange membrane water electrolyzers. Adv. Energy Mater..

[20] Wang X, Zhong H, Xi S, Lee WSV, Xue J (2022). Understanding of oxygen redox in oxygen evolution reaction. Adv. Mater..

[21] Pan Y (2020). Direct evidence of boosted oxygen evolution over perovskite by enhanced lattice oxygen participation. Nat. Commun..

[22] Zhang K, Zou R (2021). Advanced transition metal-based OER electrocatalysts: current status, opportunities, and challenges. Small.

[23] Huang Z-F (2019). Chemical and structural origin of lattice oxygen oxidation in Co–Zn oxyhydroxide oxygen evolution electrocatalysts. Nat. Energy.

[24] Guan D (2020). Utilizing ion leaching effects for achieving high oxygen-evolving performance on hybrid nanocomposite with self-optimized behaviors. Nat. Commun..

[25] Kasian O, Grote JP, Geiger S, Cherevko S, Mayrhofer KJJ (2018). The common intermediates of oxygen evolution and dissolution reactions during water electrolysis on iridium. Angew. Chem. Int. Ed..

[26] Aizaz Ud Din M, Irfan S, Dar SU, Rizwan S (2020). Synthesis of 3D IrRuMn sphere as a superior oxygen evolution electrocatalyst in acidic environment. Chem. Eur. J..

[27] Yao Y (2019). Engineering the electronic structure of single atom Ru sites via compressive strain boosts acidic water oxidation electrocatalysis. Nat. Catal..

[28] Tian Y (2020). A Co-doped nanorod-like RuO_2_ electrocatalyst with abundant oxygen vacancies for acidic water oxidation. iScience.

[29] Li Y (2020). 2D intrinsically defective RuO_2_/graphene heterostructures as All-pH efficient oxygen evolving electrocatalysts with unprecedented activity. Nano Energy.

[30] Chen S (2019). Mn-doped RuO_2_ nanocrystals as highly active electrocatalysts for enhanced oxygen evolution in acidic media. ACS Catal..

[31] Hao S (2020). Dopants fixation of Ruthenium for boosting acidic oxygen evolution stability and activity. Nat. Commun..

[32] Sun J (2018). A facile strategy to construct amorphous spinel-based electrocatalysts with massive oxygen vacancies using ionic liquid dopant. Adv. Energy Mater..

[33] Zhang L (2021). Sodium-decorated amorphous/crystalline RuO_2_ with rich oxygen vacancies: a robust pH-universal oxygen evolution electrocatalyst. Angew. Chem. Int. Ed..

[34] Hwang J (2017). Perovskites in catalysis and electrocatalysis. Science.

[35] Wei ZW (2021). Reversed charge transfer and enhanced hydrogen spillover in platinum nanoclusters anchored on titanium oxide with rich oxygen vacancies boost hydrogen evolution reaction. Angew. Chem. Int. Ed..

[36] McBride, J. R., Hass, K. C., Poindexter, B. D. & Weber, W. H. Raman and X-ray studies of Ce_1__−__x_RE_x_O_2__−__y_, where RE=La, Pr, Nci, Eu, Gd, and Tb. *J. Appl. Phys*. **76**, 2435–2441 (1994).

[37] Shan J (2021). Short-range ordered iridium single atoms integrated into cobalt oxide spinel structure for highly efficient electrocatalytic water oxidation. J. Am. Chem. Soc..

[38] Preudhomme J, Tabte P (1970). Infrared studies of spinels—III: the normal II-III spinels. Spectrochim. Acta.

[39] Dupin J-C, Gonbeau D, Vinatier P, Levasseur A (2000). Systematic XPS studies of metal oxides, hydroxides and peroxides. PCCP.

[40] Yao Q (2021). A chemical etching strategy to improve and stabilize RuO_2_-based nanoassemblies for acidic oxygen evolution. Nano Energy.

[41] Kim Y (2021). Single-phase formation of Rh_2_O_3_ nanoparticles on h-BN support for highly controlled methane partial oxidation to syngas. Angew. Chem. Int. Ed..

[42] Xu S (2020). Mechanistic study of non-thermal plasma assisted CO_2_ hydrogenation over Ru supported on MgAl layered double hydroxide. Appl. Catal. B.

[43] Parastaev A (2020). Boosting CO_2_ hydrogenation via size-dependent metal–support interactions in cobalt/ceria-based catalysts. Nat. Catal..

[44] Zhang J (2019). Origin of synergistic effects in bicomponent cobalt oxide-platinum catalysts for selective hydrogenation reaction. Nat. Commun..

[45] Wang J (2021). Turning on Zn 4s electrons in a N_2_ -Zn-B_2_ configuration to stimulate remarkable ORR performance. Angew. Chem. Int. Ed..

[46] Cao D, Xu H, Cheng D (2020). Construction of defect‐rich RhCu nanotubes with highly active Rh_3_Cu_1_ alloy phase for overall water splitting in all pH values. Adv. Energy Mater..

[47] Harzandi AM (2021). Ruthenium core–shell engineering with nickel single atoms for selective oxygen evolution via nondestructive mechanism. Adv. Energy Mater..

[48] Kim J (2017). High-performance pyrochlore-type Yttrium Ruthenate electrocatalyst for oxygen evolution reaction in acidic media. J. Am. Chem. Soc..

[49] Kumari S (2017). A low-noble-metal W_1−x_Ir_x_O_3−δ_ water oxidation electrocatalyst for acidic media via rapid plasma synthesis. Energy Environ. Sci..

[50] Seitz LC (2016). A highly active and stable IrO_x_/SrIrO_3_ catalyst for the oxygen evolution reaction. Science.

[51] Yin J (2020). Iridium single atoms coupling with oxygen vacancies boosts oxygen evolution reaction in acid media. J. Am. Chem. Soc..

[52] Xiao Z (2020). Operando identification of the dynamic behavior of oxygen vacancy-rich Co_3_O_4_ for oxygen evolution reaction. J. Am. Chem. Soc..

[53] Sardar K (2014). Water-splitting electrocatalysis in acid conditions using ruthenate-iridate pyrochlores. Angew. Chem. Int. Ed..

[54] Chen T-Y (2015). Heterojunction confinement on the atomic structure evolution of near monolayer core–shell nanocatalysts in redox reactions of a direct methanol fuel cell. J. Mater. Chem. A.

[55] Sanchez Casalongue HG (2014). In situ observation of surface species on iridium oxide nanoparticles during the oxygen evolution reaction. Angew. Chem. Int. Ed..

[56] Lin C (2021). In-situ reconstructed Ru atom array on α-MnO_2_ with enhanced performance for acidic water oxidation. Nat. Catal..

[57] Man IC (2011). Universality in oxygen evolution electrocatalysis on oxide surfaces. ChemCatChem.

[58] Zagalskaya A, Alexandrov V (2020). Role of defects in the interplay between adsorbate evolving and lattice oxygen mechanisms of the oxygen evolution reaction in RuO_2_ and IrO_2_. ACS Catal..

[59] Dickens CF, Nørskov JK (2017). A theoretical investigation into the role of surface defects for oxygen evolution on RuO_2_. J. Phys. Chem. C..

[60] Hafner J (2008). Ab-initio simulations of materials using VASP: density-functional theory and beyond. J. Comput. Chem..

[61] Perdew JP, Burke K, Ernzerhof M (1996). Generalized gradient approximation made simple. Phys. Rev. Lett..

[62] Perdew JP (1998). Burke, K. & Ernzerhof, M. Perdew, Burke, and Ernzerhof reply. Phys. Rev. Lett..

[63] Kresse G, Furthmiiller J (1996). Efficiency of ab-initio total energy calculations for metals and semiconductors using a plane-wave basis set. Comput. Mater. Sci..

[64] Kresse G (1996). Efficient iterative schemes for ab initio total-energy calculations using a plane-wave basis set. Phys. Rev. B Condens. Matter.

[65] Stoerzinger KA (2017). Orientation-dependent oxygen evolution on RuO_2_ without lattice exchange. ACS Energy Lett..

[66] Nørskov JK, Rossmeisl J, Logadottir A, Lindqvist L (2004). Origin of the overpotential for oxygen reduction at a fuel-cell cathode. J. Phys. Chem. B.

[67] Guo C (2021). Toward computational design of chemical reactions with reaction phase diagram. WIREs Comput. Mol. Sci..

[68] Guo P, Fu X, Deak P, Frauenheim T, Xiao J (2021). Activity and mechanism mapping of photocatalytic NO_2_ conversion on the anatase TiO_2_(101) surface. J. Phys. Chem. Lett..

[69] Li A (2022). Enhancing the stability of cobalt spinel oxide towards sustainable oxygen evolution in acid. Nat. Catal..

[70] Long J (2020). Direct electrochemical ammonia synthesis from nitric oxide. Angew. Chem. Int. Ed..

