# Supplementary material for: Unraveling oxygen vacancy site mechanism of Rh-doped RuO2 catalyst for long-lasting acidic water oxidation
Source: Nat Commun. 2023 Mar 14;14:1412. doi: 10.1038/s41467-023-37008-8 (PMC10015077; doi:10.1038/s41467-023-37008-8)
Supplement: Supplementary file 1 — Supplementary Information [file 41467_2023_37008_MOESM1_ESM.pdf]

Supplementary Information for

Unraveling oxygen vacancy site mechanism of Rh-doped RuO_2 catalyst for long-lasting acidic water oxidation

Yi Wang[1,2,3], Rong Yang[1,2,4], Yajun Ding[1,2], Bo Zhang[5], Hao Li[6,7], Bing Bai[1,2,3], Mingrun Li[1,2], Yi Cui[6,7], Jianping Xiao[1,2], and Zhong-Shuai Wu[1,2]

[1]State Key Laboratory of Catalysis, Dalian Institute of Chemical Physics, Chinese Academy of Sciences, 457 Zhongshan Road, Dalian 116023, China.

[2]Dalian National Laboratory for Clean Energy, Chinese Academy of Sciences, 457 Zhongshan Road, Dalian 116023, China

[3]University of Chinese Academy of Sciences, 19 A Yuquan Road, Shijingshan District, Beijing 100049, China

[4]Department of Chemistry, School of Science, Tianjin University, Tianjin 300072, China

[5]CAS Key Laboratory of Science and Technology on Applied Catalysis, Dalian Institute of Chemical Physics Chinese Academy of Sciences, 457 Zhongshan Road, Dalian 116023, China

[6]Vacuum Interconnected Nanotech Workstation, Suzhou Institute of Nano-Tech and Nano-Bionics, Chinese Academy of Sciences, Suzhou, 215123, China.

[7]School of Nano Technology and Nano Bionics University of Science and Technology of China, Hefei 230026, China

Corresponding author(s). E-mail(s):

xiao@dicp.ac.cn (J.P. Xiao); wuzs@dicp.ac.cn (Z.-S. Wu);

This file contains

- Supplementary Figures 1−20
- Supplementary Tables 1−10
- Supplementary Note 1−2
- References

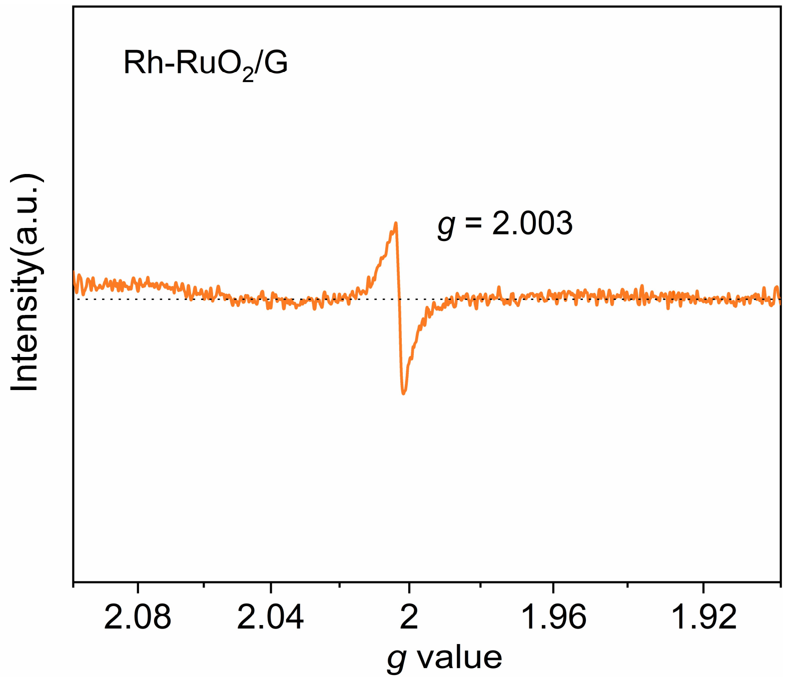

Supplementary Fig. 1 | Oxygen vacancies structure characterization. EPR spectrum of Rh-RuO_2/G.

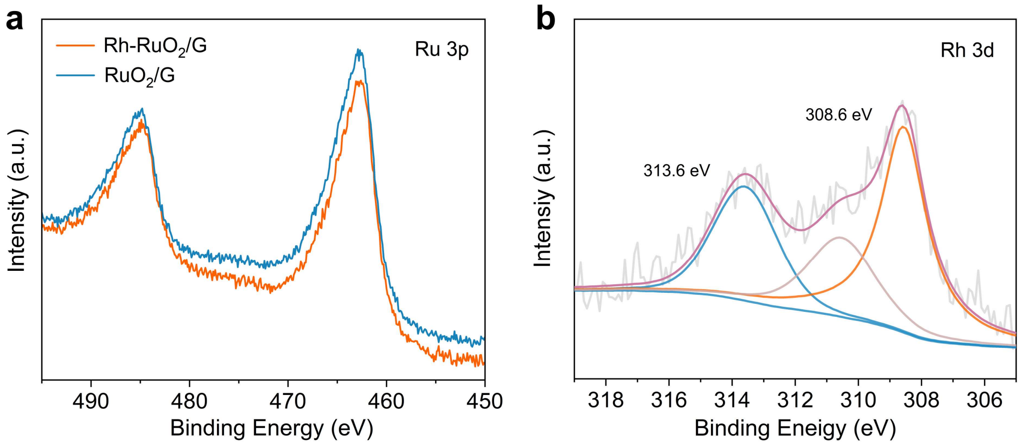

Supplementary Fig. 2 | XPS analysis of RuO_2/G and Rh-RuO_2/G. (a) *Ru 3p* XPS spectra of Rh-RuO_2/G and RuO_2/G. (b) *Rh 3d* XPS spectrum of Rh-RuO_2/G.

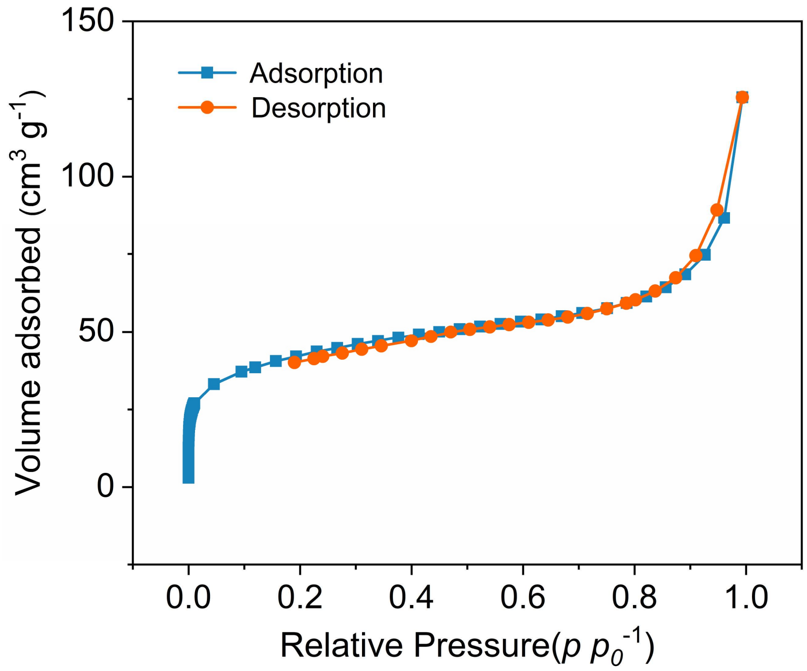

Supplementary Fig. 3 | The N_2 adsorption-desorption isotherm of Rh-RuO_2/G.

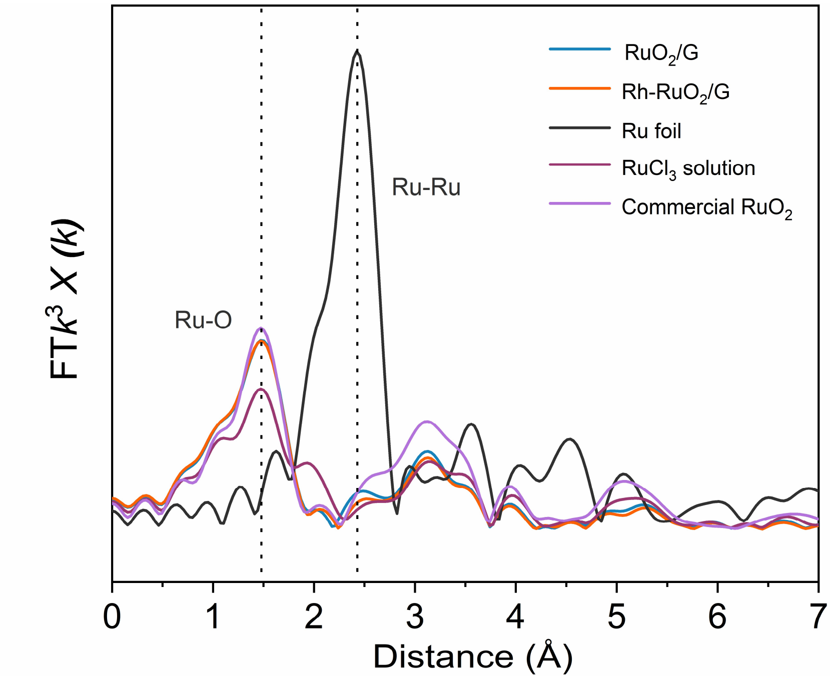

Supplementary Fig. 4 | Chemical coordination environment analysis. FT-EXAFS spectra of Ru edge for Rh-RuO_2/G, RuO_2/G, Ru foil, $RuCl_3$ solution and commercial RuO_2.

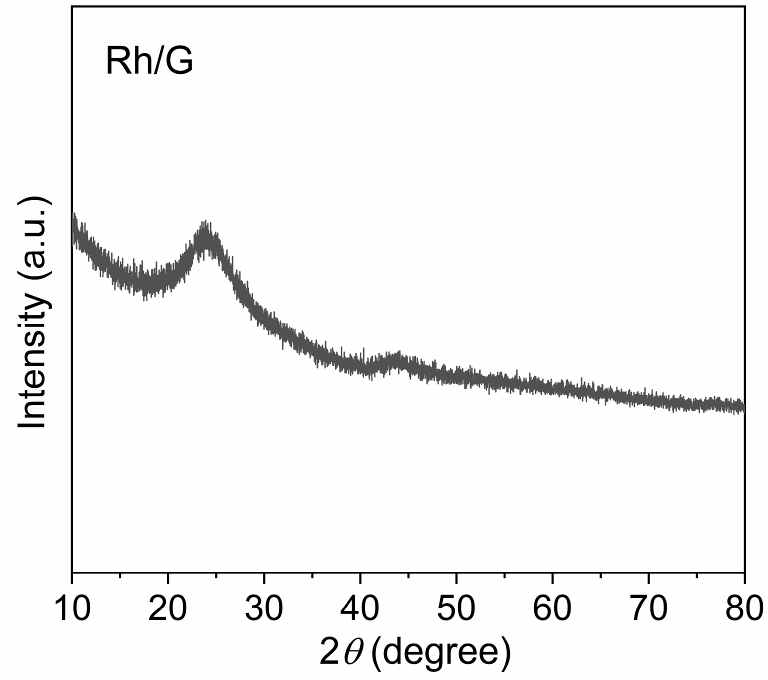

Supplementary Fig. 5 | XRD pattern of Rh/G. The two broad diffraction peaks at approximately 23.6º and 43.6º are derived from the graphene oxide substrates.

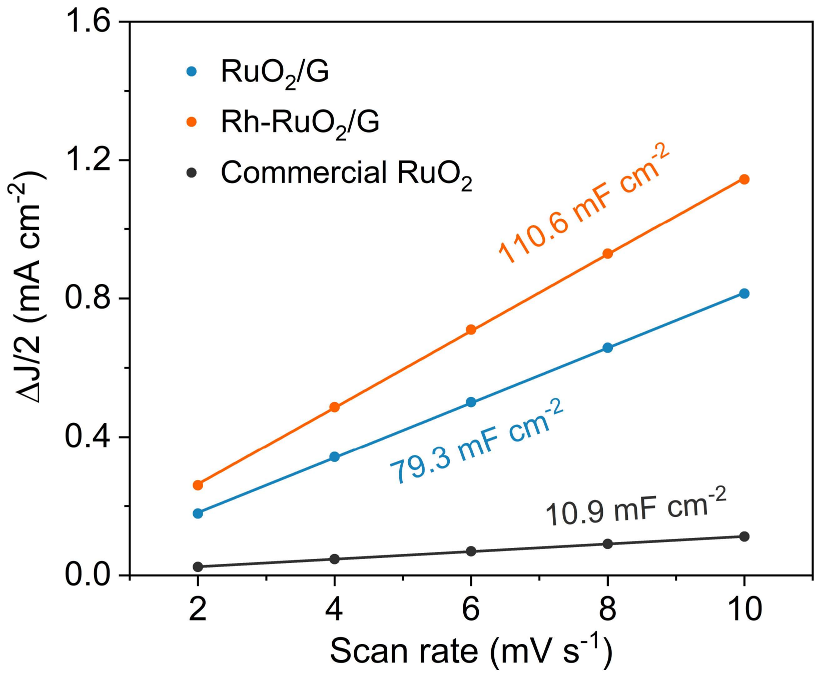

Supplementary Fig. 6 | Electrochemically active surface areas analysis for acidic OER. Capacitive current at 0.96 V of Rh-RuO_2/G, RuO_2/G, and commercial RuO_2 as a function of scan rate.

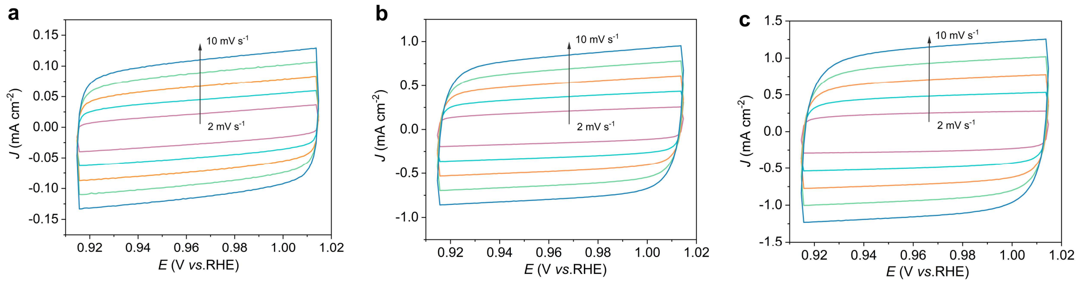

Supplementary Fig. 7 | Electrochemically active surface areas analysis for acidic OER. CV curves of (a) commercial RuO_2, (b) RuO_2/G and (c) Rh-RuO_2/G catalysts in the non-Faradaic region in acid solution, obtained at different scanning rates.

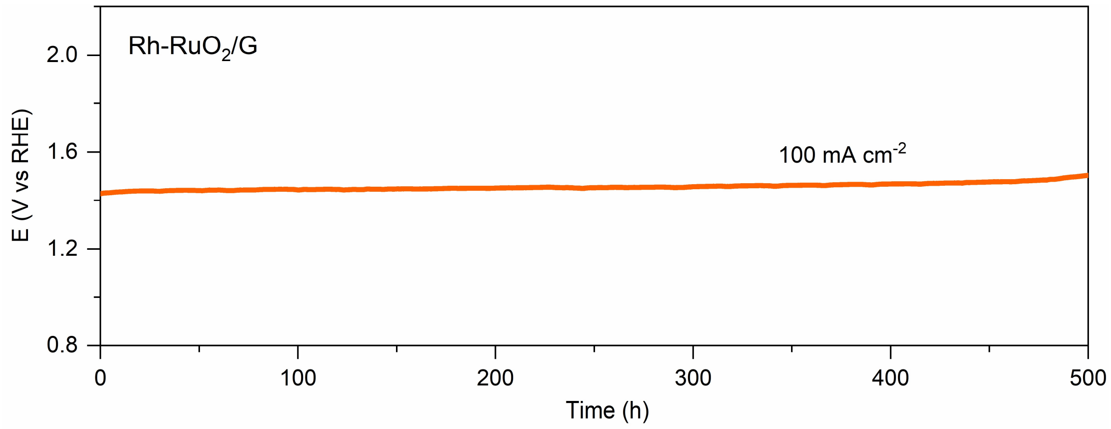

Supplementary Fig. 8 | Electrochemical stability evaluation for acidic OER. The galvanostatic curves of Rh-RuO_2/G at a current density of 100 mA cm^{-2}.

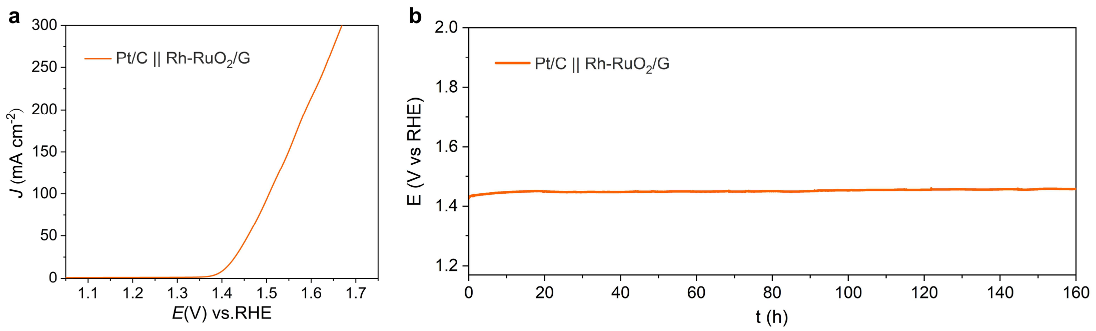

Supplementary Fig. 9 | Significantly enhanced overall water splitting performance of full device. (a) Polarization curve and (b) galvanostatic curves at a current density of 10 mA cm^{-2} of Pt/C || Rh-RuO_2/G for overall water splitting in 0.5 M H_2SO_4.

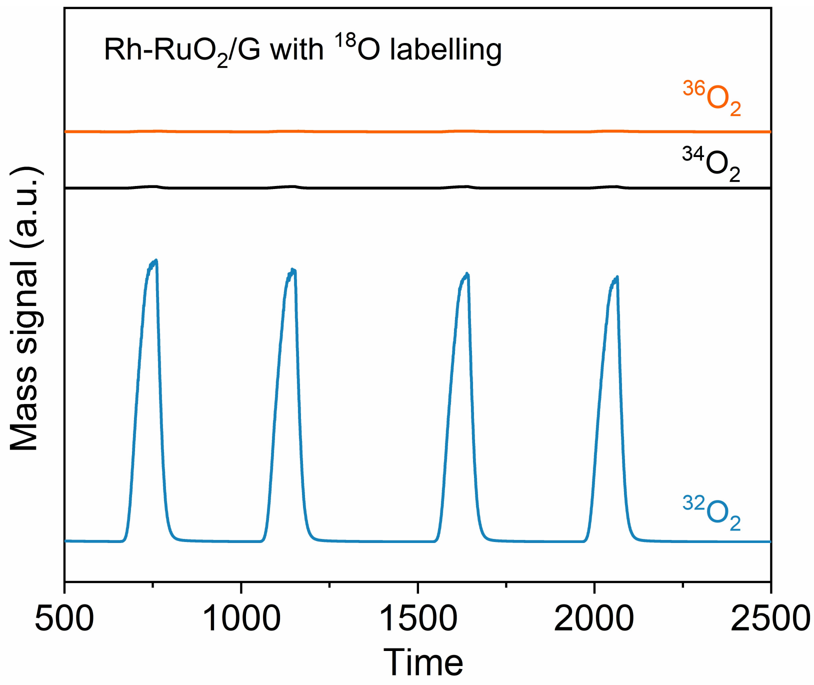

Supplementary Fig. 10 | OER mechanism analysis. Operando DEMS signals of $^{36}O_2$, $^{34}O_2$ and $^{32}O_2$ for Rh-RuO_2/G with ^{18}O labelling in $H_2{}^{16}O$ aqueous sulfuric acid electrolyte within four times of LSV at 1.1–1.9 V (*vs.* RHE).

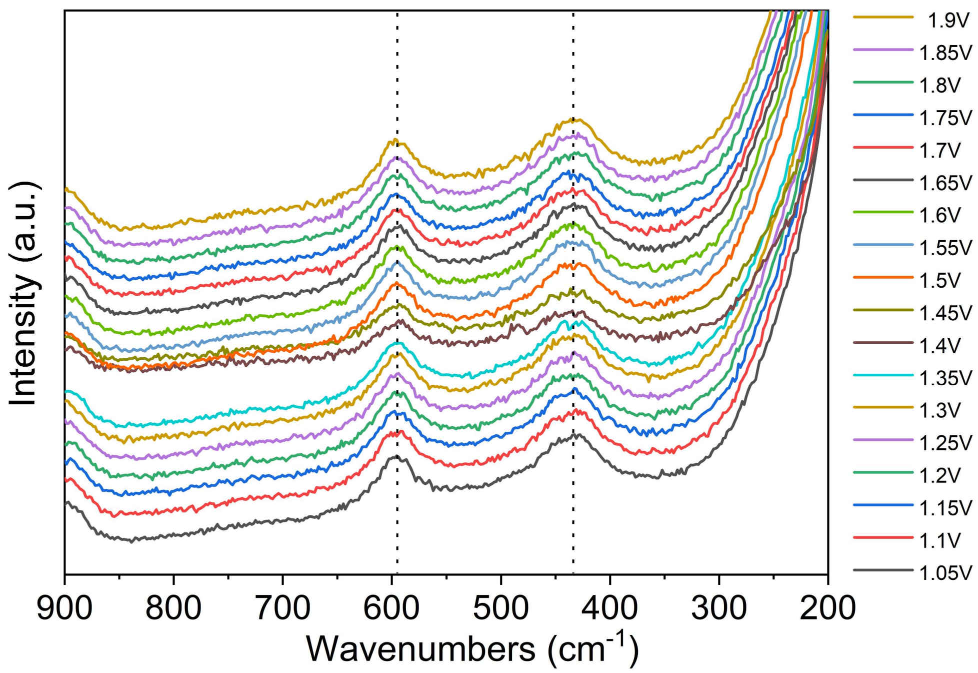

Supplementary Fig. 11 | Crystal structure stability analysis during OER. Operando Raman spectra recorded of the resultant Rh-RuO_2/G during the multi-potential steps (1.05−1.9 V).

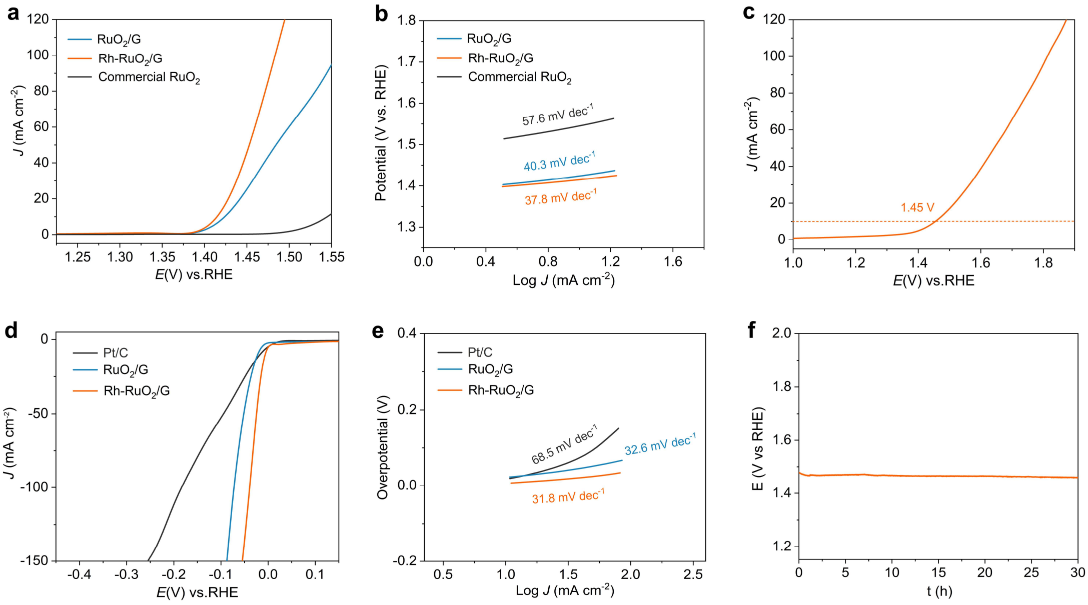

Supplementary Fig. 12 | Electrocatalytic performance of Rh-RuO_2/G, RuO_2/G, and commercial RuO_2 catalysts in 1 M KOH solution with 95% *iR* compensation. (a,b) LSV polarization curves and Tafel plots for OER. (c) LSV curve of the Rh-RuO_2/G in 1 M KOH in a two-electrode system. (d,e) LSV polarization curves and Tafel plots for HER. (f) Chronopotentiometry curve of water electrolysis at 10 mA cm^{-2}.

Supplementary Note 1 Rechargeable Li-O_2 battery.

The Li-O_2 battery was assembled with a prepared cathode (16 mm diameter), a glass fiber separator (Whatman), 70 μL electrolyte (1.0 M lithium bis(trifluoromethanesulfonyl)imide in tetraethylene glycol dimethyl ether) and a Li foil anode in an Ar filled glove box (H_2O < 0.01 ppm, O_2 < 0.01 ppm). The cathode was prepared by brush coating the slurry of obtained catalysts (Rh-RuO_2/G, RuO_2/G, commercial RuO_2, or graphite carbon) with polyvinylidene fluoride (weight ratio is 9:1) onto carbon paper, then dried at 120 °C under vacuum for 12 h. The Li-O_2 batteries were tested as 2032 coin-type configuration with holes on the cathode side. Then the cells were tested in 1 atm O_2 atmosphere (H_2O < 0.1 ppm) using a CT3001A type battery testing system (LAND electronics Co. Ltd., Wuhan, China) at current density of 200 mA g^{-1} with a cut-off specific capacity of 1000 mAh g^{-1}. The current density and specific capacity were calculated based on the mass of catalysts.

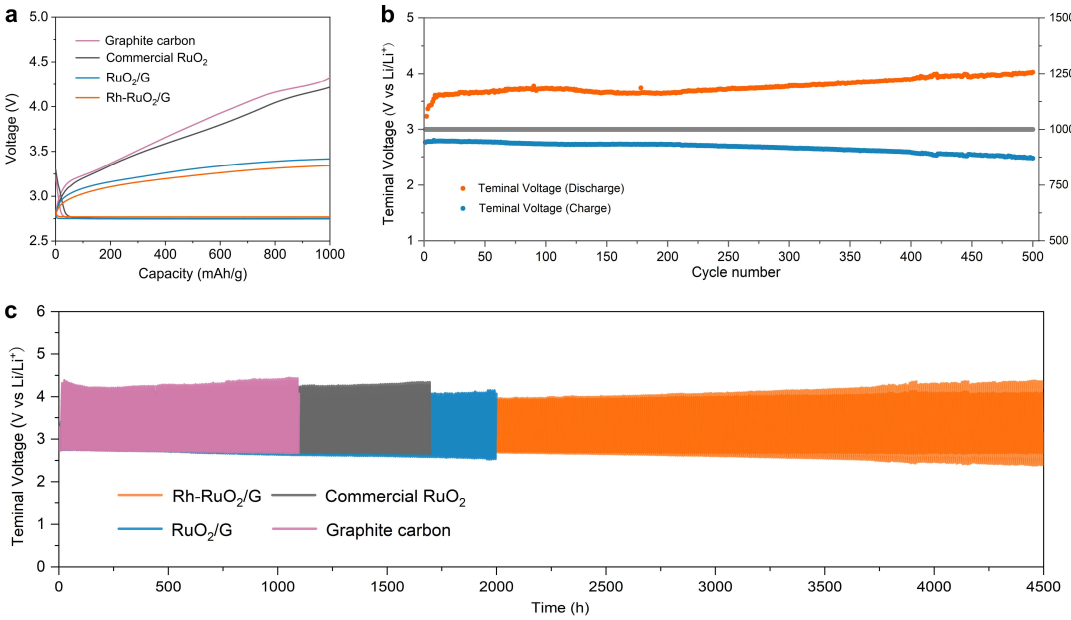

Supplementary Fig. 13 | Electrochemical performance of Li-O_2 battery based on Rh-RuO_2/G cathode at a current density of 200 mA g^{-1} and a fixed capacity of 1000 mAh g^{-1}. (a) Full first discharge/charge profiles of Li-O_2 batteries based on Rh-RuO_2/G, RuO_2/G, commercial RuO_2 and graphite carbon catalysts. (b) Cycling stability and terminal discharge-charge of Rh-RuO_2/G cathode for Li-O_2 batteries. (c) Discharge/charge cycling stability of Li-O_2 batteries based on Rh-RuO_2/G, RuO_2/G, commercial RuO_2 and graphite carbon catalysts, tested at a current density of 200 mA g^{-1} and a fixed specific capacity of 1000 mAh g^{-1}.

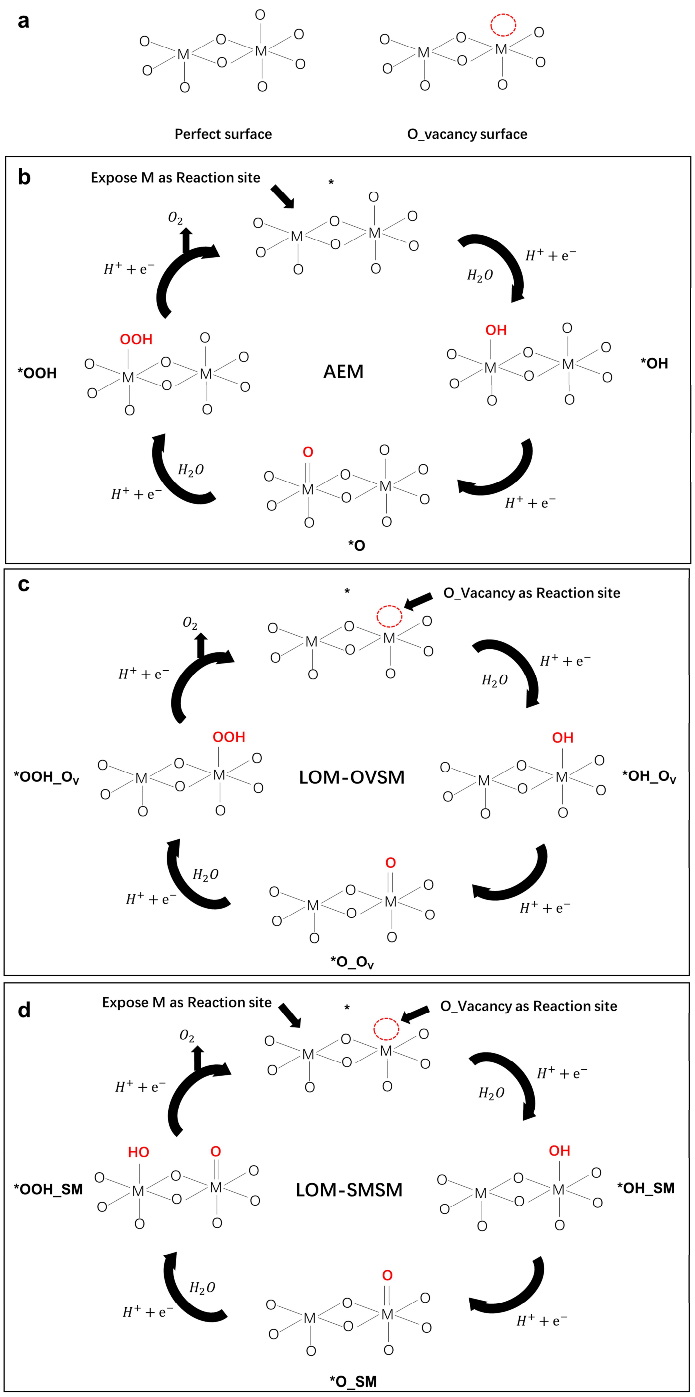

Supplementary Fig. 14 | The OER mechanisms with the different local configurations. (a) The configuration of perfect and defect structure. (b) Proposed AEM mechanism. (c) Proposed LOM-OVSM mechanism. (d) Proposed LOM-SMSM mechanism (the metal site is Ru or Rh). The naming of each intermediate is written next to the corresponding structure (*e.g.*, *OH, *O and *OOH).

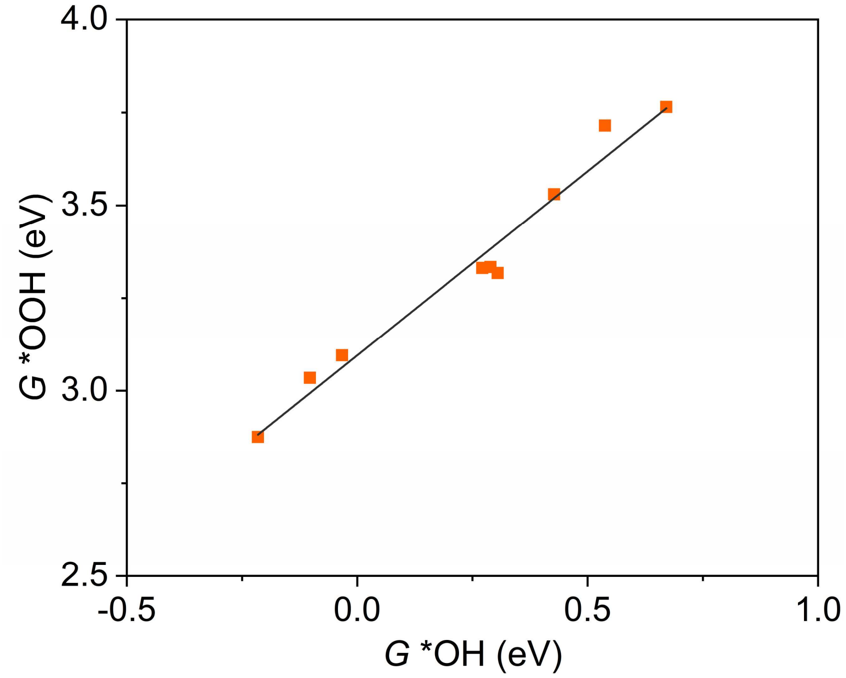

Supplementary Fig. 15 | The scaling relationship for AEM mechanism. The scaling relationship between adsorption free energies of *OH and *OOH.

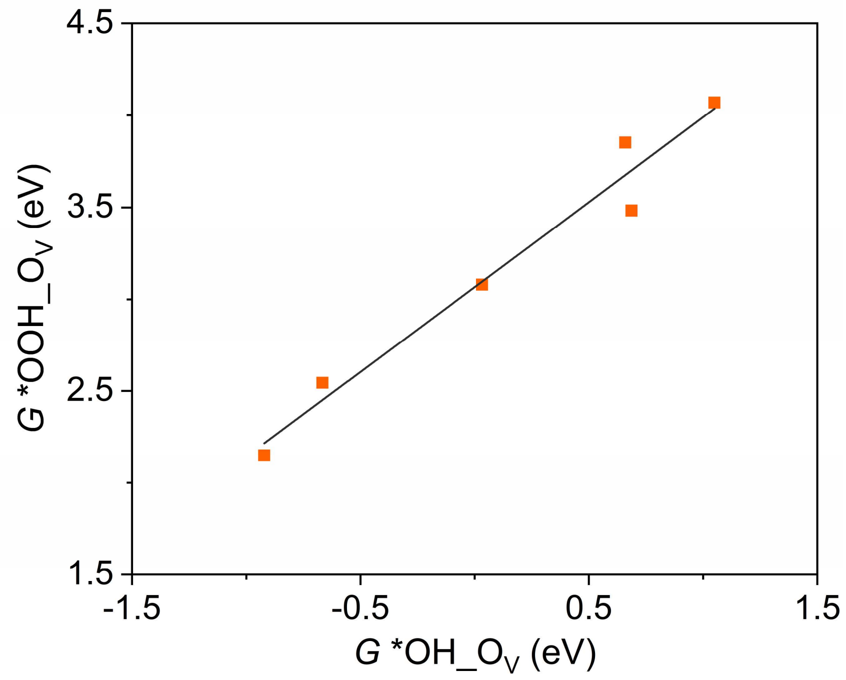

Supplementary Fig. 16 | The scaling relationship for LOM-OVSM mechanism. The scaling relationship between adsorption free energies of *OOH_O$_V$ and *OH_O$_V$.

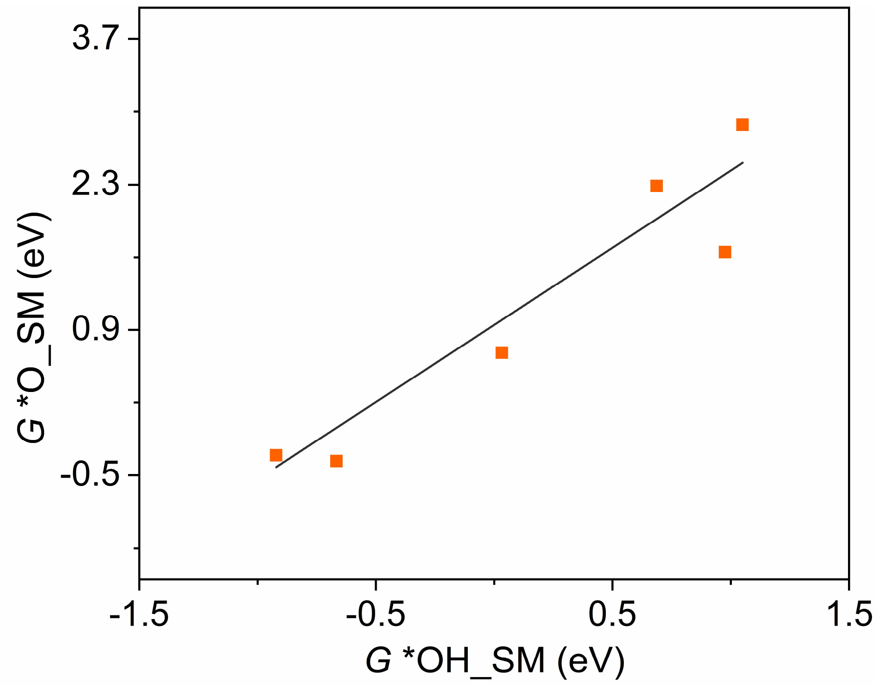

Supplementary Fig. 17 | The scaling relationship for LOM-SMSM mechanism. The scaling relationship between adsorption free energies of *O_SM and *OH_SM.

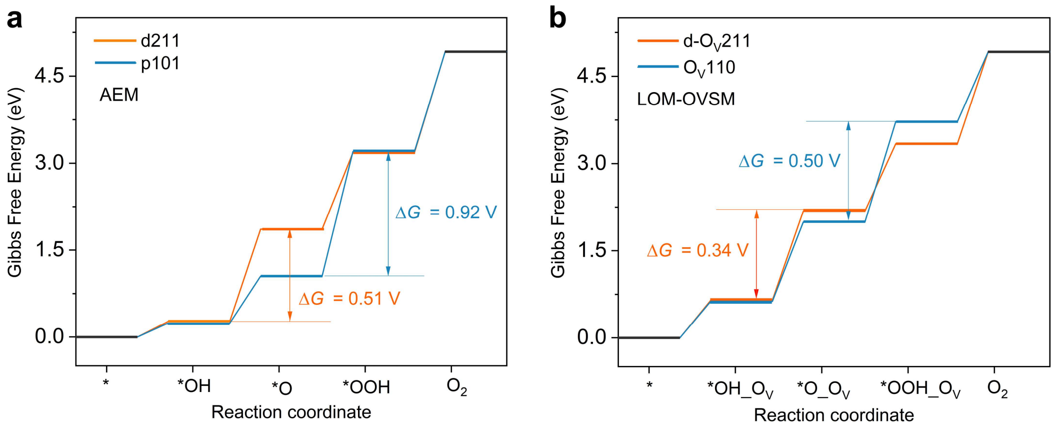

Supplementary Fig. 18 | Reaction free energy diagram for OER. Reaction free energies of the (a) AEM mechanism with the best OER performance on perfect RuO_2(101) (p101) and Rh-RuO_2(211) (d211). (b) LOM-OVSM mechanism with the best OER performance on defect RuO_2(110) (O_V110) and Rh-RuO_2(211) (d-O_V211).

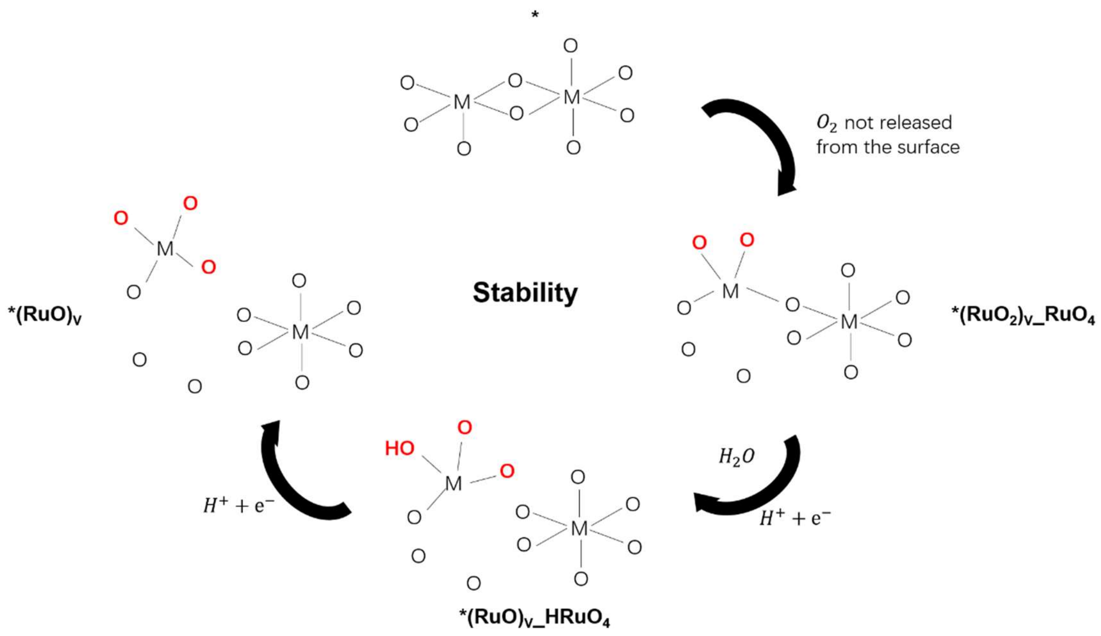

Supplementary Fig. 19 | The mechanism of the RuO_2-like surface resolving. For the stability of perfect RuO_2, perfect Rh-RuO_2, defect RuO_2 and defect Rh-RuO_2 can be described by their resolving. The simplest of dissolution mechanisms investigated at the perfect Rh-RuO_2 and defect Rh-RuO_2. Unreleased O_2 binds to surface metals, such as Ru, may simply rotate and form an adsorbed RuO_4-like species, breaking an Ru−O bond. The species desorbs into solution phase, breaking a second Ru−O bond along the row from which it came. Subsequent H adsorption and deprotonation at the two resulting surface oxygen vacancies can be assumed to be facile under OER conditions. The naming of each intermediate is written next to the corresponding structure (*e.g.*, *(RuO_2)$_V$_RuO_4, *(RuO)$_V$_$HRuO_4$ and *(RuO)$_V$).

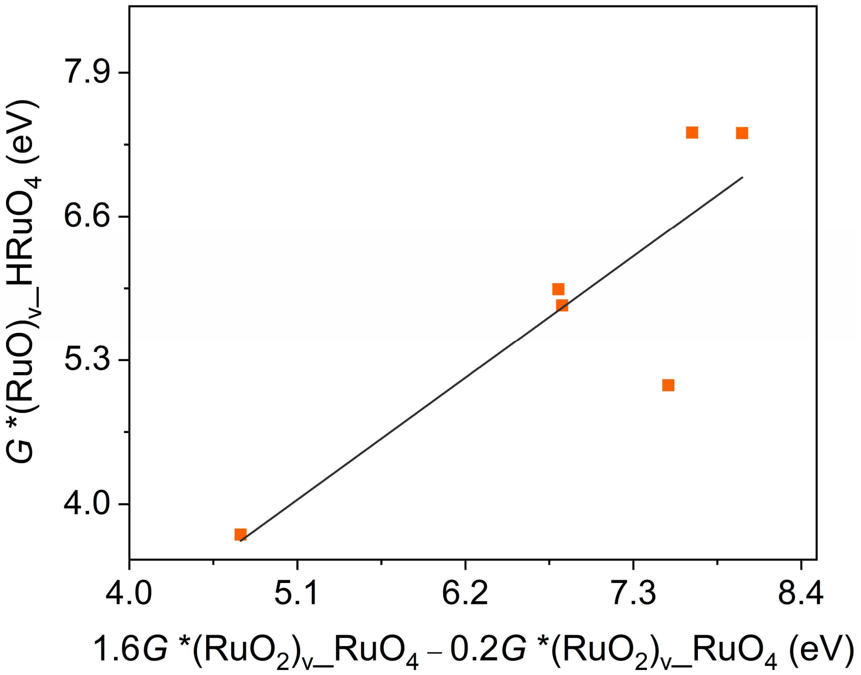

Supplementary Fig. 20 | The scaling relationships for resolving mechanism between adsorption free energies of involved intermediate species and adsorption free energy of $*RuO_4$ over perfect Rh-RuO_2 and defect Rh-RuO_2.

Supplementary Table 1 | ICP-AES results of Rh-RuO_2/G

Elements	Ru	Rh
**Weight (mg cm^{-2})**	0.0064	0.00044
**Weight %**	20.16	1.38

Supplementary Table 2 | EXAFS fitting parameters of Ru K-edge for all samples

Sample	Shell	N	R (Å)	σ^2 (10^{-2} Å^2)	ΔE_0 (eV)	r-factor (%)
Ru Foil	Ru−Ru	12.0	2.67	-	-	-
Commercial RuO_2	Ru−O	6.0	1.97	-	-	-
Rh-RuO_2/G	Ru−O	5.7	1.97	0.6	3.7	0.03
Rh-RuO_2/G (at 1.5 V)	Ru−O	5.5	1.97	0.6	5.5	0.05
RuO_2/G	Ru−O	5.7	1.97	0.6	3.8	0.03
RuO_2/G (at 1.5 V)	Ru−O	5.5	1.97	0.6	4.4	0.02

The data range used for data fitting in k-space (Δk) and R-space (ΔR) are 3.0−12.5 Å^{-1} and 1.0−2.0 Å, respectively.

Supplementary Table 3 | Performance comparison of Rh-RuO_2/G with the reported Ru-based OER catalysts

Catalyst	η_{10} (mV vs.RHE)	Stability performance	Electrolyte	Refs.
**Rh-RuO_2/G**	161	700h @50 mA cm^{-2}	0.5 M H_2SO_4	This work
**12Ru/MnO_2**	161	200h @10 mA cm^{-2}	0.1 M $HClO_4$	1
**Ru_1-Pt_3Cu**	220	28h @10 mA cm^{-2}	0.1 M $HClO_4$	2
**$Sn_{0.1}$-RuO_2@NCP**	178	150h @10 mA cm^{-2}	0.5 M H_2SO_4	3
**Ni-Ru@RuO_x-HL**	184	200h @20 mA cm^{-2}	0.5 M H_2SO_4	4
**Ru/RuS_2**	201	24h @10 mA cm^{-2}	0.5 M H_2SO_4	5
**Mn-RuO_2**	158	10h @10 mA cm^{-2}	0.5 M H_2SO_4	6
**RuB_2**	223	10h @10 mA cm^{-2}	0.5 M H_2SO_4	7
**RuIr-NC**	165	122h @1 mA cm^{-2}	0.05 M H_2SO_4	8
**Zn/RuO_2**	206	30h @10 mA cm^{-2}	0.5 M H_2SO_4	9
**$CaCuRu_4O_{12}$**	171	24h @10 mA cm^{-2}	0.5 M H_2SO_4	10
**$Y_2Ru_2O_{7-\delta}$**	190	8h @1 mA cm^{-2}	0.1 M $HClO_4$	11
**$Cr_{0.6}Ru_{0.4}O_2$**	178	10h @10 mA cm^{-2}	0.5 M H_2SO_4	12
**RuRh@$(RuRh)O_2$**	245	8000s @5 mA cm^{-2}	0.1 M $HClO_4$	13
**3D IrRuMn**	260	8h @10 mA cm^{-2}	0.1 M $HClO_4$	14
**IrRu@Te**	220	20h @10 mA cm^{-2}	0.5 M H_2SO_4	15
**RuIr@CoNC**	223	40h @10 mA cm^{-2}	0.5 M H_2SO_4	16
**RuO_2 NS**	199	20000s @10 mA cm^{-2}	0.5 M H_2SO_4	17
**Ru/Fe ONAs**	238	9h @5 mA cm^{-2}	0.5 M H_2SO_4	18
**$W_{0.2}Er_{0.1}Ru_{0.7}O_{2-\delta}$**	168	500h @10 mA cm^{-2}	0.5 M H_2SO_4	19
**$RuNi_2$@G-250**	227	24h @10 mA cm^{-2}	0.5 M H_2SO_4	20

Supplementary Table 4 | Summary of every structure exist mechanism in OER

	AEM			AEM	LOM-O_V	LOM-Ru		
p101	√		O_V101	√	√	√		
p110	×		O_V110	×	√	×		
p211	√		O_V211	√	√	√		
	AEM-Ru	AEM-Rh		AEM-Ru	AEM-Rh	LOM-O_V	LOM-Ru	LOM-Rh
d101	×	×	d-O_V101	√	×	√	√	√
d110	×	×	d-O_V110	×	×	√	×	√
d211	√	√	d-O_V211	√	√	√	√	√

Note: Perfect RuO_2 and Rh-RuO_2 only exist AEM mechanism, after involving oxygen vacancy format to defect RuO_2 and Rh-RuO_2 would promote another LOM mechanism.

Supplementary Table 5 | Formation energies of different surfaces after doping Rh of RuO_2

Surface	Formation energy (eV Å^{-2})
d(101)	-2.82
d(110)	-2.40
d(211)	0.08

Supplementary Note 2 Formation energy of doping Rh surfaces.

The thermodynamic stability of doping Rh surfaces can be confirmed by the formation energies:

$$\Delta E(\text{surface}) = \frac{E_{\text{t}}(\text{Ru}_{\text{N-x}}\text{Rh}_{\text{x}}\text{O}_{\text{2N}}) - \text{x}E(\text{Rh}_{\text{bulk}}) + \text{x}E(\text{Ru}_{\text{bulk}}) - E_{\text{t}}(\text{Ru}_{\text{N}}\text{O}_{\text{2N-x}}) - \frac{1}{2}\text{x}E(\text{O}_2)}{\text{x}}$$

where $E_{\text{t}}(\text{Ru}_{\text{N-x}}\text{RhO}_{\text{2N}})$, $E(\text{Rh}_{\text{bulk}})$, $E(\text{Ru}_{\text{bulk}})$, $E_{\text{t}}(\text{Ru}_{\text{N}}\text{O}_{\text{2N-x}})$, and $E(\text{O}_2)$ correspond to the electronic energies of x doping Rh RuO_2 (note as $\text{Ru}_{\text{N-x}}\text{Rh}_{\text{x}}\text{O}_{\text{2N}}$) slab, Rh bulk, Ru bulk, a RuO_2 include x oxygen vacancies (note as $\text{Ru}_{\text{N}}\text{O}_{\text{2N-x}}$) slab, and gas-phase O_2, and A represent the area of slab respectively. For different surfaces Rh doping formation energy shown in Supplementary Table 5, for different amount Rh doping in the (101) shown in Supplementary Table 6.

Supplementary Table 6 | Formation energies of different amount Rh doping in RuO_2(101)

Surface	Formation energy (eV Å^{-2})
$Ru_{N-1}RhO_{2N}$	-2.82
$Ru_{N-2}Ru_2O_{2N}$	-2.19
$Ru_{N-3}Ru_3O_{2N}$	-0.26

Supplementary Table 7 | The corrections of zero-point energy and entropy of adsorbed species. All energies are in eV. (*T* = 298.15 K)

Species	*ZPE*	*TS*	*ZPE-TS*
* OH	0.36	0.08	0.28
* O	0.08	0.05	0.03
* OOH	0.46	0.12	0.34
* O_Ov	0.09	0.03	0.05
* OH_Ov	0.38	0.06	0.32
* OOH_Ov	0.36	0.08	0.28
* O_SM	0.38	0.06	0.32
* OH_SM	0.09	0.03	0.05
* OOH_SM	0.36	0.08	0.28

Supplementary Table 8 | The OER performance on various sites of RhO_2 clusters

Substrate	Cluster	Site	Overpotential (V vs. RHE)
**Perfect RuO_2 (101)**	Rh_6O_6	Site1	0.47
		Site2	0.52
	Rh_6O_{12}	Site1	1.93
		Site2	1.67
**Defect RuO_2 (101)**	Rh_6O_6	Site1	1.32
		Site2	0.32
	Rh_6O_{12}	Site1	0.58
		Site2	0.49

Supplementary Table 9 | The structures of various RhO_2 clusters and substrates. The grey, green, red color balls represent the Ru, Rh and O atoms. Specially, the yellow balls represent two reaction active sites, namely site1 and site2.

Substrates / Clusters	Perfect RuO_2(101)	Defect RuO_2(101)
Rh_6O_6	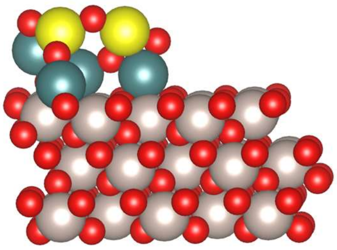	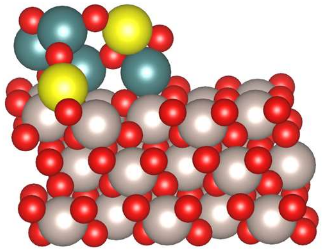
Rh_6O_{12}	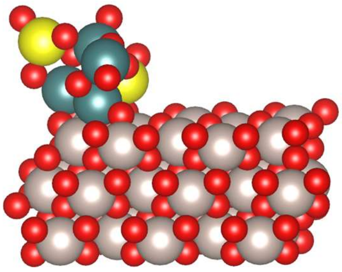	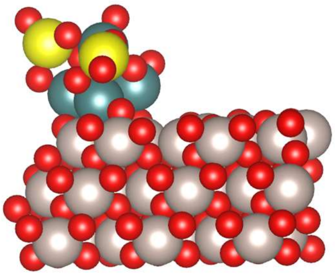

Supplementary Table 10 | Formation energies of different surfaces after doping Rh of RuO_2 using the RhO_2 and RuO_2 phases as the reference for Rh and Ru

Surfaces	Pure RuO_2 (meV Å^{-2})	Pure RhO_2 (meV Å^{-2})	Rh doped RuO_2 (meV Å^{-2})
**(101)**	166	191	168
**(110)**	220	239	222
**(211)**	296	333	298

Supplementary References

1. Lin, C. *et al.* In-situ reconstructed Ru atom array on α-MnO_2 with enhanced performance for acidic water oxidation. *Nat. Catal.* **4**, 1012−1023 (2021).

2. Yao, Y. *et al.* Engineering the electronic structure of single atom Ru sites via compressive strain boosts acidic water oxidation electrocatalysis. *Nat. Catal.* **2**, 304−313 (2019).

3. Qiu, L., Zheng, G., He, Y., Lei, L. & Zhang, X. Ultra-small Sn-RuO_2 nanoparticles supported on N-doped carbon polyhedra for highly active and durable oxygen evolution reaction in acidic media. *Chem. Eng. J.* **409**, 128155 (2021).

4. Harzandi, A.M. *et al.* Ruthenium core–shell engineering with nickel single atoms for selective oxygen evolution via nondestructive mechanism. *Adv. Energy Mater.* **11**, 2003448 (2021).

5. Zhu, J. *et al.* Regulative electronic states around ruthenium/ruthenium disulphide heterointerfaces for efficient water splitting in acidic media. *Angew. Chem. Int. Ed.* **60**, 12328−12334 (2021).

6. Chen, S. *et al.* Mn-doped RuO_2 nanocrystals as highly active electrocatalysts for enhanced oxygen evolution in acidic media. *ACS Catal.* **10**, 1152−1160 (2019).

7. Chen, D. *et al.* Ionothermal route to phase-pure RuB_2 catalysts for efficient oxygen evolution and water splitting in acidic media. *ACS Energy Lett.* **5**, 2909−2915 (2020).

8. Wu, D. *et al.* Efficient overall water splitting in acid with anisotropic metal nanosheets. *Nat. Commun.* **12**, 1145 (2021).

9. Zhang, H., Wu, B., Su, J., Zhao, K. & Chen, L. MOF-derived zinc-doped ruthenium

oxide hollow nanorods as highly active and stable electrocatalysts for oxygen evolution in acidic media. *ChemNanoMat* **7**, 117−121 (2021).

10. Miao, X. *et al.* Quadruple perovskite ruthenate as a highly efficient catalyst for acidic water oxidation. *Nat. Commun.* **10**, 3809 (2019).

11. Kim, J. *et al.* High-performance pyrochlore-type yttrium ruthenate electrocatalyst for oxygen evolution reaction in acidic media. *J. Am. Chem. Soc.* **139**, 12076−12083 (2017).

12. Lin, Y. *et al.* Chromium-ruthenium oxide solid solution electrocatalyst for highly efficient oxygen evolution reaction in acidic media. *Nat. Commun.* **10**, 162 (2019).

13. Wang, K. *et al.* Ultrathin RuRh@(RuRh)O_2 core@shell nanosheets as stable oxygen evolution electrocatalysts. *J. Mater. Chem. A* **8**, 15746−15751 (2020).

14. Aizaz Ud Din, M., Irfan, S., Dar, S.U. & Rizwan, S. Synthesis of 3D IrRuMn sphere as a superior oxygen evolution electrocatalyst in acidic environment. *Chemistry* **26**, 5662−5666 (2020).

15. Xu, J. *et al.* Strong electronic coupling between ultrafine iridium–ruthenium nanoclusters and conductive, acid-stable tellurium nanoparticle support for efficient and durable oxygen evolution in acidic and neutral media. *ACS Catal.* **10**, 3571−3579 (2020).

16. Xu, J. *et al.* Atomic-step enriched ruthenium–iridium nanocrystals anchored homogeneously on MOF-derived support for efficient and stable oxygen evolution in acidic and neutral media. *ACS Catal.* **11**, 3402−3413 (2021).

17. Zhao, Z.L. *et al.* Boosting the oxygen evolution reaction using defect-rich ultra-thin

ruthenium oxide nanosheets in acidic media. *Energy Environ. Sci.* **13**, 5143−5151 (2020).

18. Yao, Q. *et al.* A chemical etching strategy to improve and stabilize RuO_2-based nanoassemblies for acidic oxygen evolution. *Nano Energy* **84**, 105909 (2021).

19. Hao, S. *et al.* Dopants fixation of ruthenium for boosting acidic oxygen evolution stability and activity. *Nat. Commun.* **11**, 5368 (2020).

20. Cui, X. *et al.* Robust interface Ru centers for high-performance acidic oxygen evolution. *Adv. Mater.* **32**, 1908126 (2020).
